# A novel multiple time-frequency sequential coding strategy for hybrid brain-computer interface

**DOI:** 10.3389/fnhum.2022.859259

**Published:** 2022-07-29

**Authors:** Zan Yue, Qiong Wu, Shi-Yuan Ren, Man Li, Bin Shi, Yu Pan, Jing Wang

**Affiliations:** ^1^Institute of Robotics and Intelligent Systems, Xi'an Jiaotong University, Xi'an, China; ^2^Beijing Tsinghua Changgeng Hospital, Tsinghua University, Beijing, China

**Keywords:** hybrid brain-computer interface, steady-state visual evoked potentials, omitted stimulus potential, multiple time-frequencies sequential coding, event-related potential (ERP)

## Abstract

**Background:**

For brain-computer interface (BCI) communication, electroencephalography provides a preferable choice due to its high temporal resolution and portability over other neural recording techniques. However, current BCIs are unable to sufficiently use the information from time and frequency domains simultaneously. Thus, we proposed a novel hybrid time-frequency paradigm to investigate better ways of using the time and frequency information.

**Method:**

We adopt multiple omitted stimulus potential (OSP) and steady-state motion visual evoked potential (SSMVEP) to design the hybrid paradigm. A series of pre-experiments were undertaken to study factors that would influence the feasibility of the hybrid paradigm and the interaction between multiple features. After that, a novel Multiple Time-Frequencies Sequential Coding (MTFSC) strategy was introduced and explored in experiments.

**Results:**

Omissions with multiple short and long durations could effectively elicit time and frequency features, including the multi-OSP, ERP, and SSVEP in this hybrid paradigm. The MTFSC was feasible and efficient. The preliminary online analysis showed that the accuracy and the ITR of the nine-target stimulator over thirteen subjects were 89.04% and 36.37 bits/min.

**Significance:**

This study first combined the SSMVEP and multi-OSP in a hybrid paradigm to produce robust and abundant time features for coding BCI. Meanwhile, the MTFSC proved feasible and showed great potential in improving performance, such as expanding the number of BCI targets by better using time information in specific stimulated frequencies. This study holds promise for designing better BCI systems with a novel coding method.

## Introduction

Compared with conventional assistive communication technologies, brain-computer interface (BCI) establishes a new pathway between the human brain and the external environment, thereby assisting people with severe motor disabilities to control external devices or re-establish communication (Wolpaw et al., 2002; Chaudhary et al., 2016). Among various neural recording techniques such as magnetoencephalogram (MEG) and electrocorticography (ECoG), electroencephalography (EEG) has attracted the most attention due to its advantages in high temporal resolution, portability, and low cost. The earliest EEG-BCI applications were time-modulated visual evoked potential (VEP) (t-VEP)-based BCIs represented by the event-related potential (ERP) like P300. The transient time-locked response to visual stimulus used to be extracted as the control command (Farwell and Donchin, 1988; Donchin et al., 2000). However, further development of traditional t-VEP-based BCI has been limited by its deficits, such as the “repetition blindness” phenomenon and weak features that could be influenced by mental load or be easily contaminated by background noise (Squires et al., 1977; Salvaris and Sepulveda, 2009; Yin et al., 2013a). On the other hand, frequency-modulated VEP (f-VEP)-based BCIs represented by steady-state visual evoked potential (SSVEP) (which have been on focused more), provide a more robust response than t-VEP-based BCIs (Bin et al., 2009a; De Neeling and Van Hulle, 2019). However, most f-VEP studies adopted the frame-based “on/off” flickering stimulation method, in which only frequencies that are the aliquot number of the refresh rate could be used for coding (Yin et al., 2013b, 2015; Allison et al., 2014; Combaz and Van Hulle, 2015; Wang et al., 2015; Katyal and Singla, 2020). Subsequent studies proposed frequency-approximation approaches to overcome this problem, in which adopting sampled sinusoidal stimulation to create frequencies approximated to the ideal stimulation frequency (Wang and Jung, 2010; Xie et al., 2012; Chen et al., 2014a). These sampled visual presentations could be the time of the flickering frame or additional information, such as the phase of luminance or the motion of the stimulation targets. However, because of the limited range of frequencies that could evoke detectable SSVEP and the influence of harmonic components, the number of available stimulation frequencies is still lacking (Herrmann, 2001; Wang et al., 2006). Thus, BCIs purely based on f-VEP or t-VEP do not support widespread practical usage (Squires et al., 1977; Wang et al., 2006; Salvaris and Sepulveda, 2009; Chen et al., 2015; Singla and Jatana, 2017).

Continued exploration of the coding strategy is essential for the development of the BCI communication system. This is similar to the progress in mobile communication, in which hybrid coding methods like the Orthogonal Frequency-Division Multiple Access replaced the non-hybrid coding method using single time or frequency information such as the Time Division Multiple Address (TDMA) and the Frequency Division Multiple Access (FDMA) (Chen et al., 2018). Parallel endeavors also happened in VEP-based BCI field. Recently, hybrid BCI coding methods such as frequency-phase modulation and multi-frequency coding have shown great potential or outstanding performance compared with that based on f-VEP or t-VEP (Mukesh et al., 2005; Jia et al., 2010; Edlinger and Guger, 2012; Zhang et al., 2012; Hwang et al., 2013; Yin et al., 2013a,b, 2015; Allison et al., 2014; Chang et al., 2014, 2016; Chen et al., 2014a, 2015, 2017, 2018; Fan et al., 2014; Combaz and Van Hulle, 2015; Wang et al., 2015; Katyal and Singla, 2020; Xu et al., 2020). The time-frequency mixing modulation method is another one among these novel methods, in which the frequency and time information is implemented into the stimuli together. However, this method has received relatively less attention in recent years despite its importance and potential in coding information from two major domains (time and frequency) than other hybrid methods.

On the one hand, some related research adopted BCIs called the sequential BCIs, in opposition to the simultaneous BCIs (Pfurtscheller et al., 2010). In sequential methods, time and frequency information were simply added together with the cost of stimulus duration, while the information for communication per unit time did not increase (Edlinger and Guger, 2012; Fan et al., 2014). On the other hand, although some further research adopted simultaneous BCIs to elicit P300 and SSVEP simultaneously, they did not show much improvement (Chen et al., 2014a, 2015; Chang et al., 2016). These research studies focused on the combination of P300 and SSVEP, but their respective defects remain unresolved (Squires et al., 1977; Salvaris and Sepulveda, 2009; Yin et al., 2013a,b, 2015; Allison et al., 2014; Combaz and Van Hulle, 2015; Wang et al., 2015; Katyal and Singla, 2020). Besides, the P300 and SSVEP features evoked in these studies were mainly realized by simultaneously adopting two different visual presentation elements, such as color, shape, size, and symbol (Yin et al., 2013b, 2015; Allison et al., 2014; Wang et al., 2015; Chang et al., 2016). Thus, it could also cause adverse effects on user performance and satisfaction because of high mental workloads (Kerr, 1973; Schwent et al., 1976; Pfurtscheller et al., 2010; Liu and Li, 2012; Choi et al., 2017). Another defect is that these early research studies still use the flickering method of SSVEP. In long-term use, continuous light flicker and contrast changes may also cause disturbances or visual fatigue (Sutter, 1992; Xie et al., 2012).

Recent research on the omitted stimulus potential (OSP, a kind of ERP) or the SSVEP-blocking shows a simple method to elicit ERP features on SSVEP stimuli. This method offers benefits by omitting events or blocking SSVEP stimuli without introducing extra visual presentation elements that may bring mental workloads (Sutter, 1992; Xu et al., 2014; Wu et al., 2016). Meanwhile, except for the SSVEP feature, an additional SSVEP-blocking feature was found to supplement the P300 feature recognition (Wu et al., 2016). However, the frequency limitation on monitoring stimulators remains settled, except for the research that adopts extra LED devices to present stimulators (Sutter, 1992). Whether recent advances in breaking through the frequency limitation of monitors, such as the frequency-approximation approaches, could be used in this time-frequency hybrid paradigm has not been explored (Wang and Jung, 2010; Xie et al., 2012; Chen et al., 2014a). Besides, in these research studies, further examination on how related detailed parameters would influence the combination of ERP and SSVEP has not been studied.

Thus, to investigate better ways of using the time and frequency information, this paper proposed a novel hybrid coding strategy to incorporate time modulated BCI with frequency modulated BCI simultaneously. We explored a hybrid BCI paradigm that combined multi-OSP and steady-state motion visual evoked potential (SSMVEP), which can simultaneously elicit multiple ERP and SSVEP features and was unrestricted by the monitors' refresh rate. A series of pre-experiments were undertaken to study the feasibility of combining single OSP and multi OSPs with the SSMVEP stimuli, and the interaction of multiple features under different factors. Afterwards a novel Multiple Time-Frequencies Sequential Coding (MTFSC) strategy was introduced to explore the hybrid time and frequency coding method. We verified the usability of this MSTFC method in experiment with a nine-target stimulator. At last, the effectiveness, limitations, and how this novel coding method would inspire other paradigms are further discussed.

## Methods

### Subjects and data acquisition

Thirteen healthy subjects (seven males, six females, aged 22–26 years) were recruited for this research, after providing informed written consent following a protocol approved by the institutional review board of Xi'an Jiaotong University. All of them had normal or corrected-to-normal vision. Subjects were seated on a comfortable chair in a lit room, keeping a viewing distance of approximately 70 cm to the monitor.

EEG signals were recorded with a 16-electrode EEG cap using the g. USBamp system (g.tec Inc., Austria) at 1,200 Hz sampling rate according to the 10-10 international system. Previous studies had shown that both SSVEP and ERP features were usually acquired around the occipital and parietal regions such as Pz, Oz and POz, while the ERP features could also be found in frontal and central regions such as Fz, Cz, and CPz (Krusienski et al., 2006; Bin et al., 2009b). Thus, similar to other hybrid ERPs and SSVEP studies, we adopted the electrodes around the above regions including O1, Oz, O2, PO7, PO3, POz, PO4, PO8, P3, Pz, P4, CP3, CPz, CP4, Cz, and Fz for our paradigm (Yin et al., 2013a, 2015; Wu et al., 2016). All channels were referenced to a bilateral mastoid and grounded to the frontal position (Fpz). EEG signals were band-pass filtered at 0.01–100Hz to remove artifacts and notch filtered between 48 and 52 Hz to remove power line interference in the online filter stage.

### Experiment design

**Figure 1:**
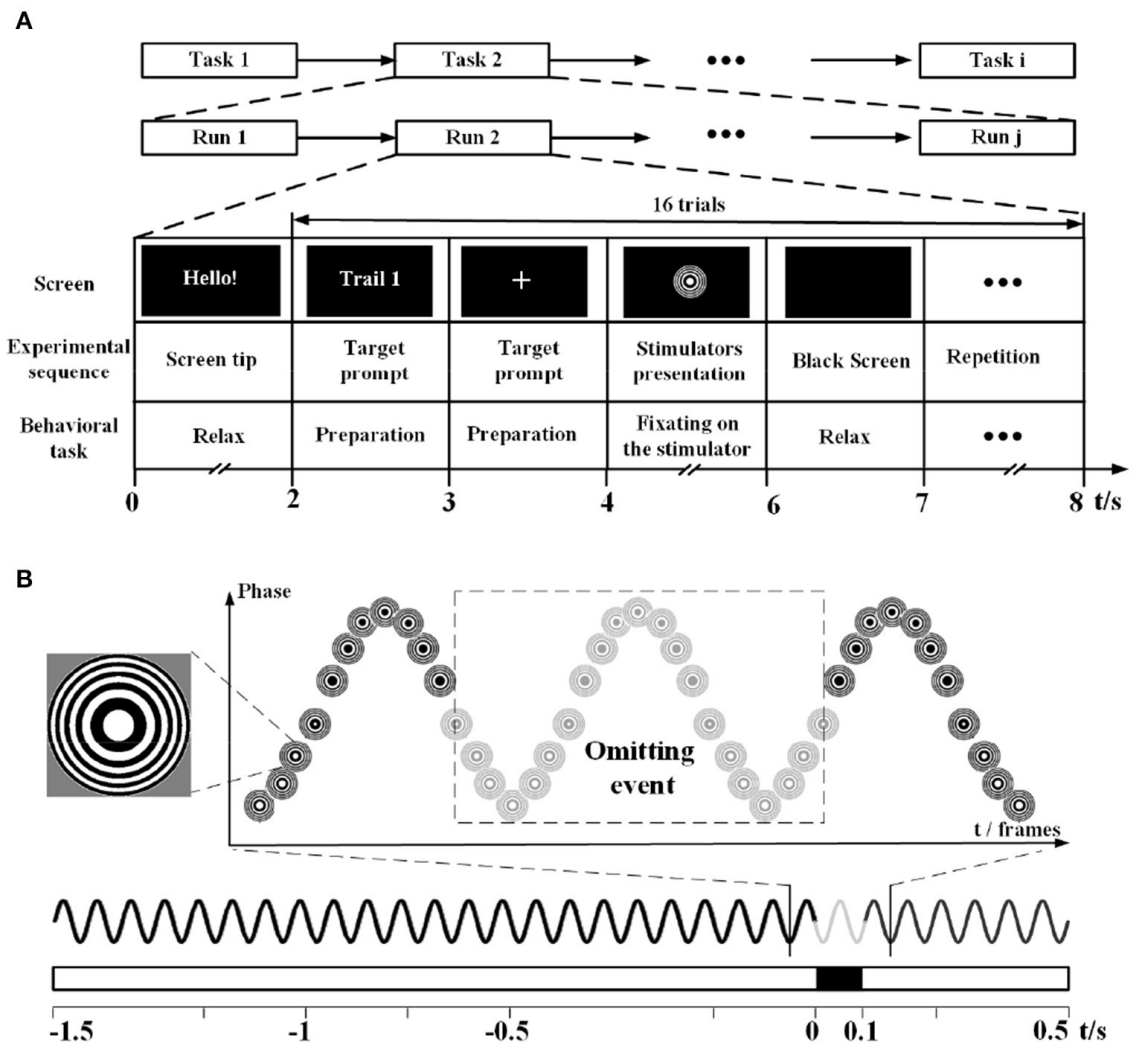
The experimental sequence and the example of stimulus sequence. **(A)** The timing of the experimental sequence and behavioral task. For different experiments, the number of task runs, stimulators, or time duration may vary. **(B)** The bottom shows two simplified presentation methods of the stimulus sequence in a waveform graph or rectangular bar. The solid black line (or the white rectangular bar) represents the SSVEP stimuli, while the solid gray line (or the black rectangular bar) represents the omitting events. The top shows how this stimulus sequence is realized by arranging the frames of Newton's ring stimulator.

The novel hybrid BCI paradigm was based on the combination of SSMVEP and different OSPs, as illustrated in Figure 1. The SSMVEP is a kind of special SSVEP evoked by the motion-based frequency-approximation approach, in which the “on*/*off” flickering frequency is replaced with the periodic movement frequency to overcome the limitation of the refresh rate (Volosyak et al., 2009; Xie et al., 2012; Chai et al., 2019; Stawicki and Volosyak, 2020). In this study, the SSMVEP stimuli were adopted from the previous Newton's Rings method, which elicited the SSVEP with the oscillating expansion and contractions called motion reversal (Xie et al., 2012). The OSP stimuli were introduced by pausing the periodic motion of the Newton's Rings. The 23 inches display used as the visual stimulator had a 60 Hz refresh rate and 1920 × 1080 screen resolution. The simulation program was developed using the Psychophysics Toolbox under MATLAB (MathWorks, Inc.).

There are three kinds of experiments included in this study: SSMVEP+ single OSP, SSMVEP+ multi OSP, and MTFSC. The first two experiments studied the feasibility and influence factors of the novel paradigm to study how the single/multi OSPs would interact with the SSMVEP, while the last experiment focused on the feasibility of the MSTFC method in offline and online BCI tasks. The exampled experimental sequence is illustrated in Figure 1A, in which the number of runs and presentation of stimulators may vary with different tasks. Each run contained a 2s relax and 16 trials. Each trial started with a 2s display of target prompt text, and the presentation of simulators, and ended with a 1 s black screen. The subsections below provide details of these experiments.

#### SSMVEP + single OSP

Considering that OSP had never been combined with the motion-based SSVEP before, we studied three factors, namely the omitting patterns, stimulation frequencies, and the duration of omission, to investigate the feasibility and the influence factors on this paradigm. The presentation of stimulators contained one Newton's ring stimulator with different frequencies or omitting factors in the center of the screen in each task. Experiments described in this subsection contained four runs in each task.

a. Omitting Patterns.
  Two tasks were included to study two types of omitting patterns during the SSMVEP stimuli: blocking and disappearing. In the blocking pattern, the SSMVEP stimuli would block at the last phase frame during the omitting event and continue the next phase frame after the omitting event, while in the disappearance pattern, Newton's Ring would disappear during the omitting course and reappear after the omitting event is finished.
  - Frequency of SSMVEP stimuli: 15 Hz.
  - Duration of omission: 0.1 s.
b. Stimulation Frequency.
  Twenty-five tasks were included to study how stimulation frequencies (from 6 Hz to 30 Hz with a step of 1 Hz) would influence the SSMVEP and the OSP features in this novel paradigm.
  - Omission pattern: disappearance pattern.
  - Duration of omission: 0.1s.
c. Duration of Omitting.
  Three tasks were designed to investigate the influences of different omission durations on the SSMVEP+OSP paradigm. Each task's omission duration was 1, 0.1, and 1/15 s (namely, one cycle of the movement of the SSMVEP stimuli). After that, an additional task with an omitting duration of 0.6 s was designed to investigate the extra potential features evoked by the reappearance of SSMVEP stimuli in this paradigm.
  - Frequency of SSMVEP stimuli: 15 Hz.
  - Omission pattern: disappearance pattern.

#### SSMVEP + multi OSPs

a. Feasibility.

To investigate whether multiple OSPs could be evoked in SSMVEP stimuli, the paradigm designed in this sub-experiment included three short and one long omitting events, respectively, with a duration of 0.1 and 0.4s. The omitting pattern used was the disappearance pattern. The whole stimulation lasted for 3 s, with a stimulation frequency of 15 Hz.

b. Order of OSP.

Two tasks corresponding to two types of OSP orders were designed to investigate whether the distinct orders would influence the paradigm. The long omitting event was arranged in the first or the second-order among all these four omitting events. The whole stimulation lasted for 3 s, while each omitting event started at 0.3 s after the preceding omission.

#### MTFSC

a. The MTFSC method.

Based on the features of the SSMVEP and multi-OSP, we introduced the novel MTFSC strategy. In this method, omitting events with different durations could be inserted into the SSVEP stimuli to expand information from frequency domain to time and frequency domain simply without introducing extra visual elements. The following equations could generate the motion phase of the stimulator with frequency f_s_ and multi-omitting events:


(1) $$\text{Stim}({\text{f}}_{\text{s}},\text{t},{\text{o}}_{\text{i}},{\text{r}}_{\text{i}})\text{=}\left\{ \begin{array}{l} \begin{array}{cc} \text{S}(\text{t})\;=\text{ cos}(\text{2}\varpi {\text{f}}_{\text{s}}\text{*}\frac{\left\lfloor \text{t}*\text{RR}\right\rfloor }{\text{RR}}), & \text{ift }\notin \left[{\text{o}}_{\text{i}},{\text{r}}_{\text{i}}\right] \end{array}\\ \text{O}(\text{t})\text{=Null},\text{ift}\in \left[{\text{o}}_{\text{i}},{\text{r}}_{\text{i}}\right] \end{array}\right.$$

Where f_*s*_ represents the stimulus frequency, RR represents the refreshing rate of the monitor, *t* is the time of the stimuli, o_*i*_ indicates the start time of *i*-th omitting event, and r_*i*_ indicates the reappearance of the SSVEP stimuli after *i*-th omitting event.

**Figure 2:**
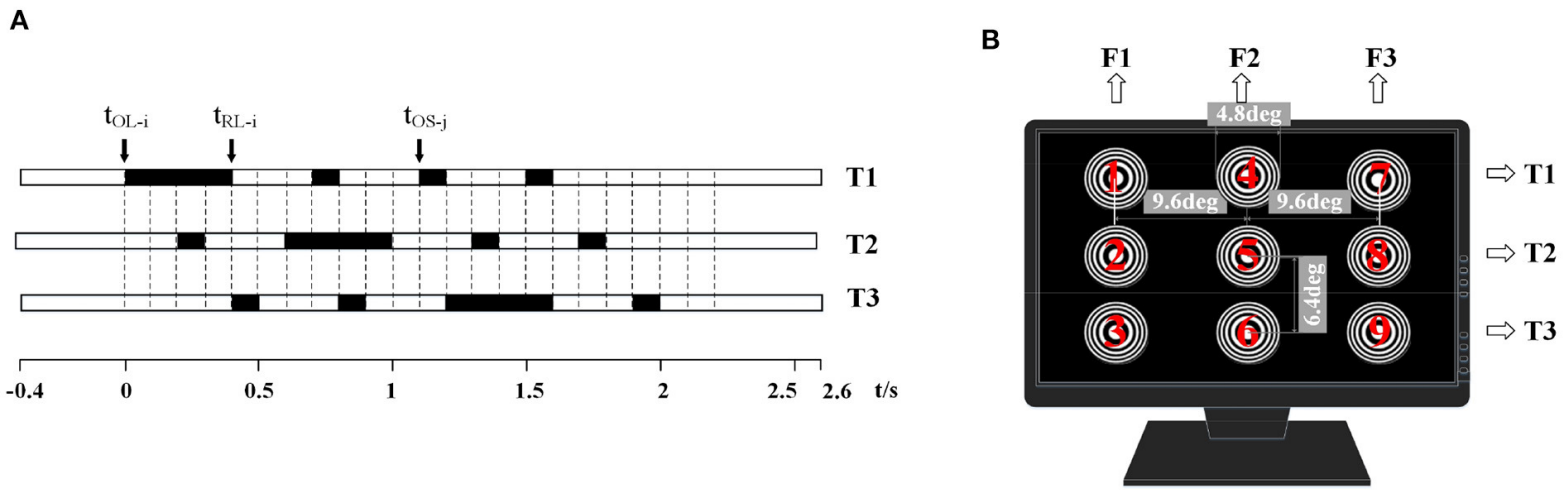
The schematic diagram of the experimental stimulators. **(A)** A kind of arrangement for omitting events was adopted in this study with one long omission (0.4 s) and three short omissions (0.1 s). O_L−i_ represents the onset time of *i*-th long omission, while the O_*Sj*_ represents the onset time of *j*-th short omission. The R_*Li*_ (R_*Sj*_) represents the end time of the i(j)-th long (short) omission, or the time of reappearance of the SSVEP stimuli after the i(j)-th omission. **(B)** Distribution of nine stimulators on the screen. The stimulators were marked 1-9 from left to right and from top to bottom. The stimulators in the same column oscillated with the same frequency, in which F1 was 5 Hz, F2 was 17 Hz, and F3 was 19 Hz. Three arrangements of omitting events differ in rows. T1 for the first row, T2 for the second row, and T3 for the third row.

When the *t* is not during the omitting events, the stimuli are represented as S(t), a sampled sinusoidal stimulation method for generating the motion phase of Newton's ring stimulator. When the *t* is during the omitting events, the stimuli omitted as the disappearance pattern, represented as O(t), namely no stimulator could be seen except the black screen. Figure 2A shows an arrangement for omission sequences adopted in this research.

b. The nine-target offline experiment.

A nine-target offline experiment explored the features of the MTFSC method. There were nine tasks corresponding to nine Newton's Rings simulators. Each task contained four runs. The presentation of stimulators is illustrated in Figure 2B. In each trial, the target prompt was displayed for 0.5 s. After that, the stimulators were presented for 3 s, followed by the dark screen for 0.5 s relaxing, then repeated until sixteen trials were completed.

c. The online experiment of 9 targets.

After that, the last online test paradigm evaluated the performance of the MTFSC application. The experiment was divided into a classifier training stage and an online test stage for every subject.

In the training stage, subjects were asked to fixate on one of the nine stimulators. Each task included two runs. The stimulus sequence was the same as in the nine-target offline experiment. There were ten runs in the online test stage. Each run's stimulus sequence was identical to the training stage, except that the dark screen was replaced with result feedback and the target prompt was displayed randomly in a trial. The reason for introducing the random presentation was for better reflection of the performance in practical application (in which the commands were commonly uncertain or random) (Chen et al., 2014a; Yin et al., 2014). The stimulators in the nine-target online experiment were in the same arrangement as the nine-target offline experiment.

### Analysis and online performance

#### Data analysis

To analyze the feature of this hybrid BCI, all the EEG data were processed offline in MATLAB. First, for pre-processing, the signals were band-pass filtered from 1 to 30 Hz (4th order zero-phase-shift Butterworth filter) to remove baseline excursion and high-frequency noises for better analysis. Previous studies have shown that evoked responses, such as the transient visual and the steady-state potentials, are time and phase-locked to the stimuli (David et al., 2006; Moratti et al., 2007; Tsoneva et al., 2015). By averaging EEG data to the identical stimuli, weak event-related potential signals could be enhanced while the background noise would decrease (Dawson, 1954). Thus, the primary time-domain information is analyzed by averaging multiple raw signal trials in each task. Then, the FFT and the topographies were analyzed to study the SSVEP features and spatial distribution. Besides, the empirical mode decomposition (EMD) method was adopted to analyze the incorporation of the ERP and OSP features, which brings more details by extracting the so-called intrinsic mode components on the nonstationary and non-linear signal (Flandrin et al., 2004; Chen et al., 2016). At last, the time-frequency was analyzed to study the interplay features of the MTFSC hybrid paradigm.

#### Template-based recognition

**Figure 3:**
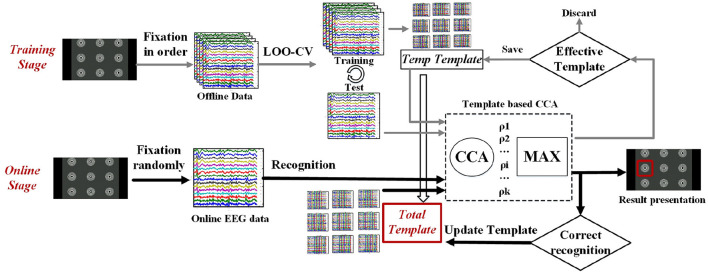
The flow chart of the recognition algorithm is based on CCA and a complex template.

Canonical correlation analysis (CCA) is a classical nonparametric multivariable analysis method. This method investigates the inherent relation between two sets of variables and found that the maximum individual template signals outperformed the standard CCA (Hotelling, 1992; Mukesh et al., 2005; Lin et al., 2006; Nakanishi et al., 2015) for more extensive BCI applications. Besides, considering the individual difference in EEG data and the potential mutual interference between the time and frequency domains in this paradigm, a template signal-based recognition would be better for the complex features in this study. Considering that the OSP and the SSVEP features are both time-locked, the average template signal would contain sufficient features for recognition. Thus, a template-based CCA was used for target identification. This study used the Leave-One-Out Cross-Validation (LOO-CV) method and the incremental learning method to generate and update the target template, as illustrated in Figure 3. Thus, the classification recognition model would depend less on long-duration offline training and be good at dynamic adaptation.

LOO-CV is a cross-validation method used to evaluate the performance of a classifier by calculating classification accuracy (Leamy and Ward, 2010). N-1 samples were used as the training set if there were N samples, while the remaining one was used as the test set. It was repeated N times, and the average classification accuracy was calculated as the number of correct classifications over N. Compared with other methods, LOO-CV can acquire reliable results closest to the original sample distribution. We employed the main idea of LOO-CV to generate a classifier in this paper. The details were as follows.

(1) According to the prompt, subjects must fixate on one of the M stimulus targets, each of which collects N groups of EEG data. After pre-processing, data is stored by M classes, and each class contains N groups.
(2) A data group is randomly taken from every class as test data, and the remaining N-1 groups are taken as training data.
(3) Training data are superimposed and averaged over N-1 times to obtain the waveform templates of each target, and the templates of M targets are built into the classifier.
(4) CCA is performed between the test data of M targets and templates. When the correlation between one group of test data and one template is maximum, the test data is identified as the same target stimulator as the template.
(5) If all M targets are recognized correctly, the classification accuracy is 100%, and the templates of M targets are the target template. Otherwise, templates are discarded.
(6) Repeat steps (2)–(5) until the iteration is 100 times. The target template is superimposed and averaged to obtain a better target classifier that stores all target waveform templates.

An incremental learning method was used during the online experiment to improve the classifier's performance (Molina, 2007). In this study, incremental learning was mainly used to improve the performance of the recognition algorithm. The recognition algorithm used here was based on the template trained from offline data with small sample size. To improve the generalization capability of the system, we used the new EEG data in the online cued experiments to update the template trained from the offline data. This was realized by adding online samples that were correctly recognized to the total template in specific weight. Thus, the recognition algorithm would be dynamically updated as the template was expanded with more information. As shown below:


(2) $$\begin{array}{l} \text{Template\_Online}=\frac{\text{Template\_Base}+\omega *\text{Online\_NewData}}{1\;+\omega } \end{array}$$

Assume that Template_Base is the classifier template generated by LOO-CV, and Online_NewData is new data generated during the online experiment. CCA was adopted between them to identify the target. When the recognition result was judged correct with a high confidence level, Online_NewData would be added to the template based on the rule of the above formula, where ω is the maximum correlation coefficient. If otherwise, it was discarded.

#### Information transfer rate

The most common measure to evaluate the BCI paradigm is information transfer rate (ITR), which is used to measure the achievable information rate per unit of time. The ITR (in bits per minute) calculation (Jia et al., 2010) is given by


(3) $$\begin{array}{l} \text{ITR}=\frac{60}{\text{T}}\left[log_{2}\text{N }+\text{ Plo}{\text{g}}_{2}\text{P }+\;\left(1\;-\text{ P}\right)\text{lo}{\text{g}}_{2}\frac{1\;-\text{ P}}{\text{N }-\;1}\right] \end{array}$$

Where T is the decision transfer interval which includes single detection time and the interval between detections, N is the number of stimulators, and P is the mean accuracy averaged over all stimulators.

## Results

### SSMVEP+ single OSP

#### Feasibility

**Figure 4:**
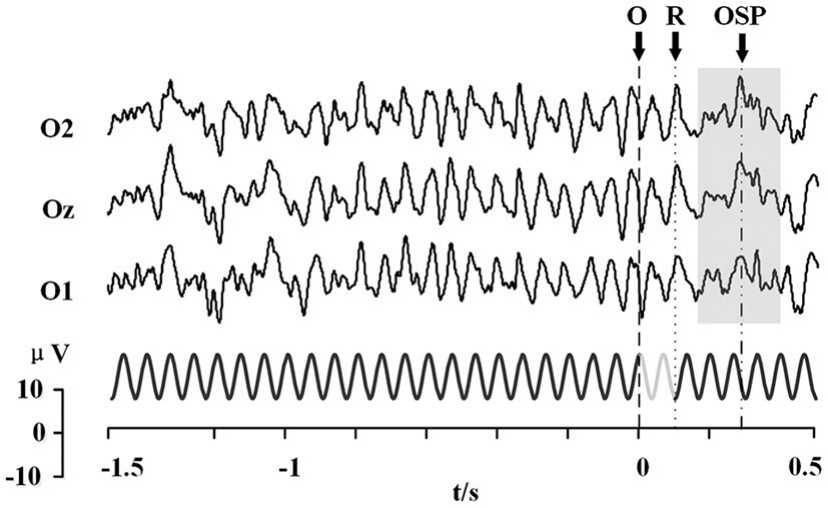
Stimulus sequence of the stimulator and the typical occipital EEG responses to the missing event on SSMVEP stimuli (64 trials). The moment at arrow “O” denotes the SSMVEP stimuli omitting, while the arrow “R” denotes the stimuli's reappearance. Gray blocks presented the primary OSP features.

As found in previous studies that combined OSP and SSVEP-blocking with SSVEP (elicited by periodic flicker stimuli), in this study too, the OSP feature was found when combined with SSMVEP (elicited by motion-based SSVEP stimuli) (Xu et al., 2013, 2014; Wu et al., 2016). Figure 4 shows the typical occipital EEG responses to the missing event on SSMVEP stimuli, averaged by 64 trials. The primary OSP feature (gray blocks) was found elicited after the omitting event (with the duration of 0.1 s). A significant positive peak after about 300 ms can be observed. A more detailed presentation of the OSP feature could be found in signal reconstruction based on EMD, as shown at the right of Supplementary Figure S1. Besides, an ERP feature evoked by the appearance of SSMVEP stimuli could also be found in the reconstruction signal (as in the left of Supplementary Figure S1). Two positive peaks are elicited in about 180 and 470 ms after the SSMVEP stimuli onset.

#### Influencing factors

a. Omitting patterns.

Both types of omitting patterns were available in eliciting the OSP feature in this sub-experiment. As shown in Supplementary Figure S2 (see Supporting Document), a positive peak at about 300 ms was found, similar to a previous study (Wu et al., 2016). This positive peak was found in the central region, such as Cz, for both the disappearance and blocking omitting patterns. However, the positive peak elicited by the disappearance omission pattern was more evident than that by the blocking pattern in Poz and Pz. Thus, to elicit a more robust OSP feature for recognition, other experiments in this study adopted the omitting pattern of disappearance.

b. Stimulation frequency.

To analyze the influence of stimulus frequency, stimulus frequencies from 6 to 30Hz were selected to study SSVEP and ERP features. After being filtered between 1 and 30 Hz, the signal was superimposed and averaged over 64 trials to eliminate irrelevant background signals. Response to different frequencies was recorded by typical electrodes Pz and POz (see Supporting Document: Supplementary Figure S3).

The OSP features could be found in all these frequencies at about 200–500 ms after the onset of omission. Similarly, the ERP features evoked by the appearance of SSMVEP stimuli could also be found in frequencies from 6 to 30 Hz, after about 100–600 ms.

The amplitude spectrum of SSVEP in different frequencies was calculated by Fast Fourier Transform at POz (see Supporting Document: Supplementary Figure S4). Most of these stimuli frequencies showed clear peaks in FFT amplitude (15 out of 25). However, the amplitude of SSVEP features was higher at frequencies of 7Hz, 10Hz, and 15–21Hz. Therefore, among these frequencies, we chose 10, 15, 16, 17, 18, 19, and 20 Hz to avoid confusion between the harmonic frequency and nearby frequency in the remaining experiments.

c. Duration of omitting.

**Figure 5:**
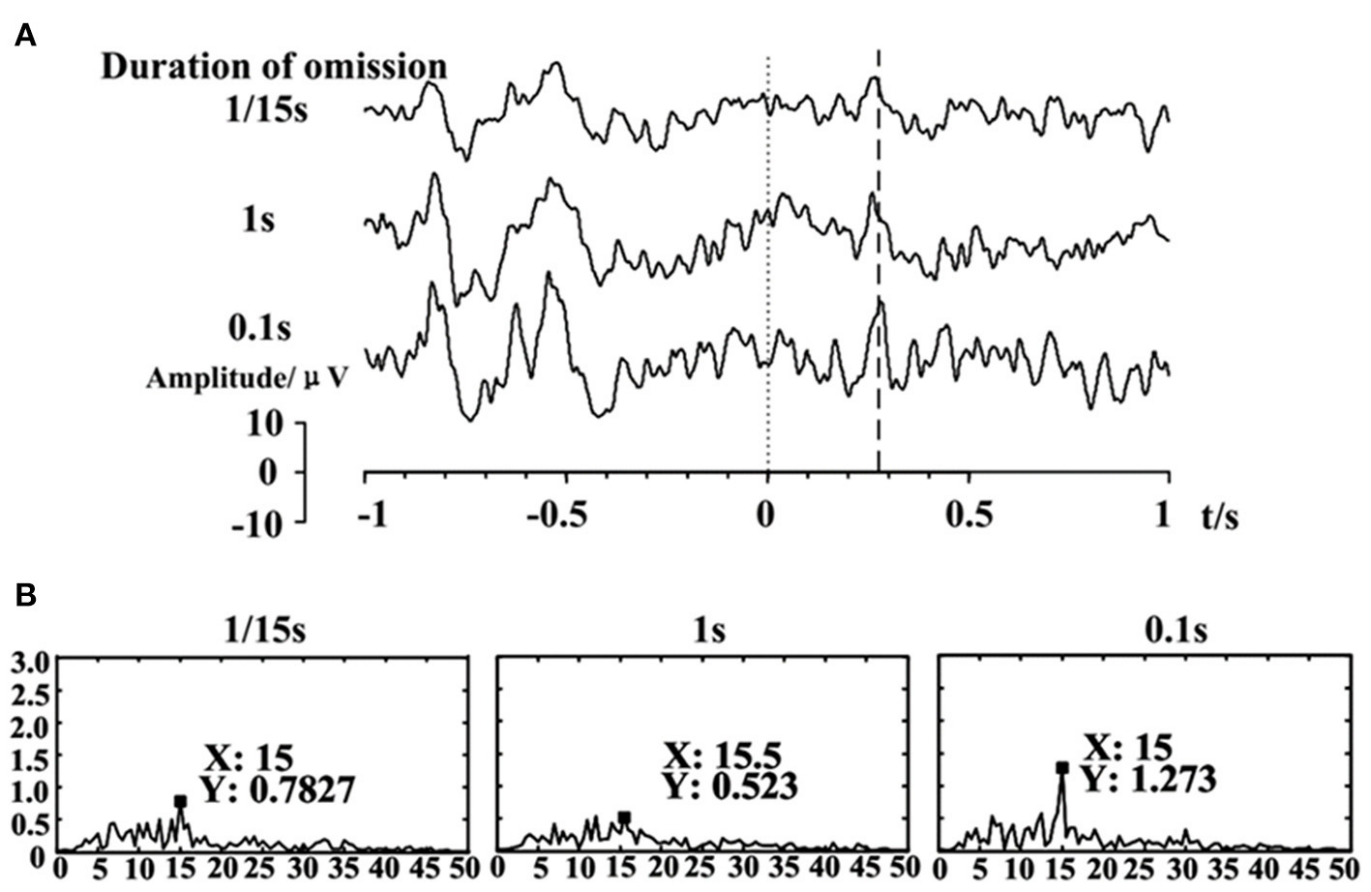
**(A)** EEG responses to different omitting durations. **(B)** The frequency analysis of the different duration of omitting at Oz electrode, with a stimulation frequency of 15 Hz.

The pre-processing data comparing the duration of omitting were superimposed and averaged over 64 trials to acquire the feature. All these durations of omission elicited OSP features, with the same positive components at about 300 ms, as in Figure 5A. It shows that the primary OSP features were not influenced by the duration of omission but only decided by the onset of the OSP in the paradigm. However, features in the frequency domain were affected by OSP when the omission duration was 1 s, with 1 s blocking of SSVEP. A longer duration of omission might negatively influence the characteristic frequencies. As shown in Figure 5B, the SSVEP frequency of 0.1 s omitting was much stronger than that of 1 s.

The omission with 1 s duration increased the time cost of the stimuli while deteriorating the frequency feature. Thus, we should avoid a too long omission duration. For the duration of 0.0667 s, it might be too short for catching the omission visually during distraction. Therefore, the omission duration of 0.1 s is a relatively superior setting for eliciting OSP features.

**Figure 6:**
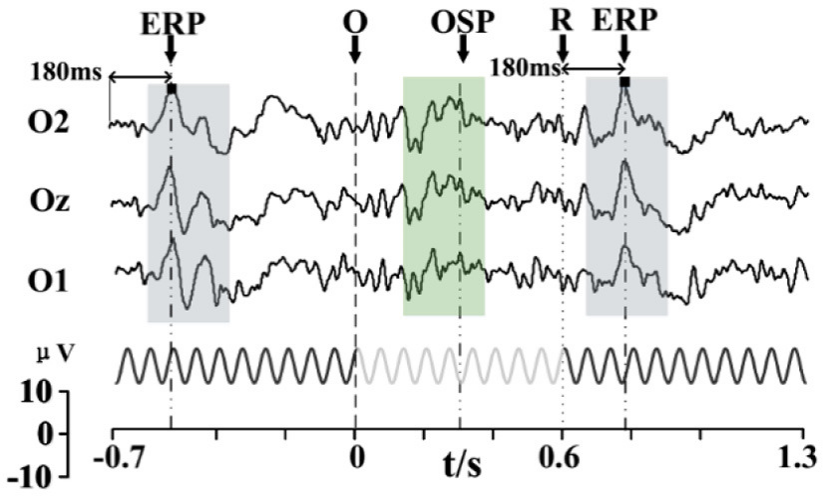
Stimulus sequence of the stimulator with 0.6 s duration, and the averaged typical EEG responses (64 trials). The green shaded area represents the OSP features, while gray shaded areas represent the main ERP features.

Considering that the onset of SSVEP stimulus can evoke ERP features, we analyzed the data in the additional experiment of 0.6 s omission to find other possible ERP features. Interestingly, except for the ERP feature that occurs after the onset of the SSMVEP stimulus, a similar ERP feature can also be observed after the reappearance of the SSMVEP stimulus. As illustrated in Figure 6, a positive peak at about 180 ms after the appearance or reappearance of the SSMVEP stimuli can be found. They both remain at the same latency, waveform, and amplitude. Therefore, omitting events with different durations might be used as additional information for coding with the ERP features. Since OSP was prominent at about 300 ms after the onset of omission, long omission could be set as 0.4 s to elicit the ERP features while avoiding the impact on ERP and shortening the single stimulus cycle.

### SSMVEP+ multi OSPs

#### Feasibility

**Figure 7:**
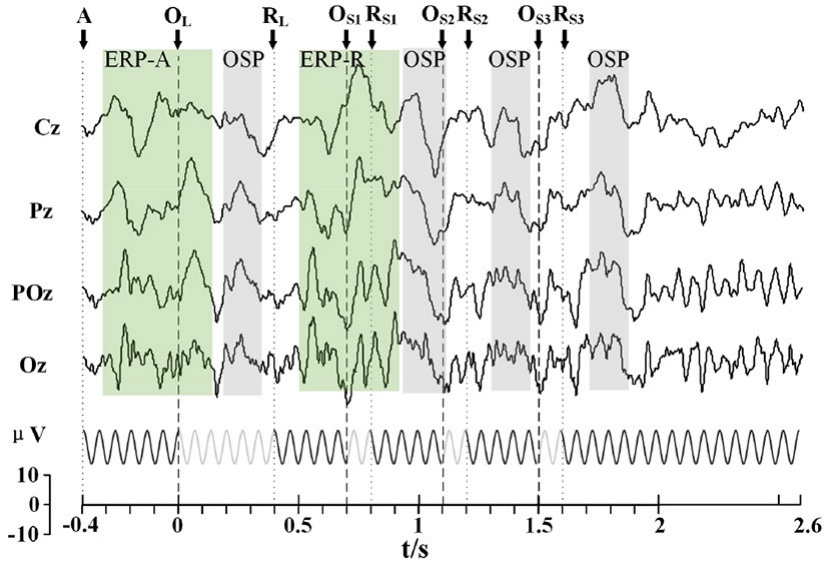
Stimulus sequence and the typical EEG response of the stimuli with four omitting events (64 trials). The Green shaded area represents the primary ERP features, while gray shaded areas represent the primary OSP features. The classical peaks of OSP and ERP features are marked with square dots.

Several segments with different omission durations were inserted in the stimulus sequence to verify whether the combination of multi OSPs and SSMVEP stimuli was workable. The typical response of ERP and OSP was unaffected by each other. All the features found in the single OSP experiments could also be found in the multi-OSP experiment. As shown in Figure 7, ERPs were elicited two times by the appearance of a stimulus in the green-shaded area, and OSPs were elicited four times by the absence of a stimulus in the gray-shaded area. Different OSP and ERP features were evoked simultaneously after combining different long and short omitting events.

#### Order of omitting events

**Figure 8:**
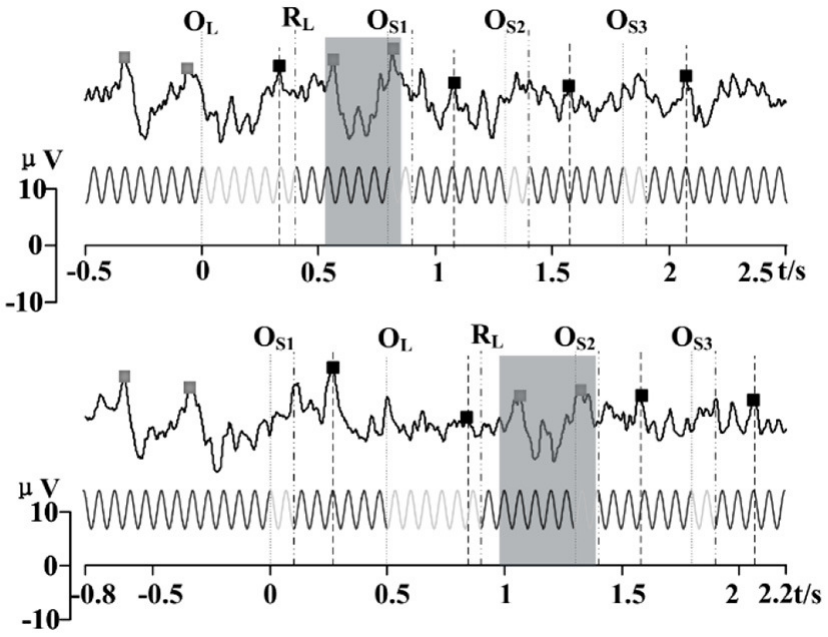
Comparison of two kinds of orders for the stimuli with multi omission (64 trials). The Gray shaded area shows a similar response after the long omitting duration in a different order. The prominent peaks of EPR and OSP are marked with square dots.

Two different orders of omitting events can elicit the same amount of two ERP and four OSP features (Figure 8). These features had corresponding sequences as the order of OSP in the paradigm and were not influenced by each other. Similar to the single omission experiment, the primary feature of OSP occurred before 300 ms after the omitting event. The ERP occurred before 500 ms after the (re)appearance of SSMVEP stimuli. Considering this, the arrangement of 300 ms's interval between omitting events was sufficient for the paradigm. Thus, the order of omitting events could be used as additional information to code the paradigm.

### MTFSC

Studies on the combination of SSMVEP and OSP inspired the novel MTFSC method. To demonstrate how the features were integrated and assess the preliminary performance of the paradigm designed with the MTFSC method, a nine-target paradigm generated by the MTFSC method was experimented offline to analyze the ERP, OSP, and SSVEP features and their interplay. Then, the nine-target paradigm was experimented online to study its performance.

#### Features

a. ERP and OSP features.

**Figure 9:**
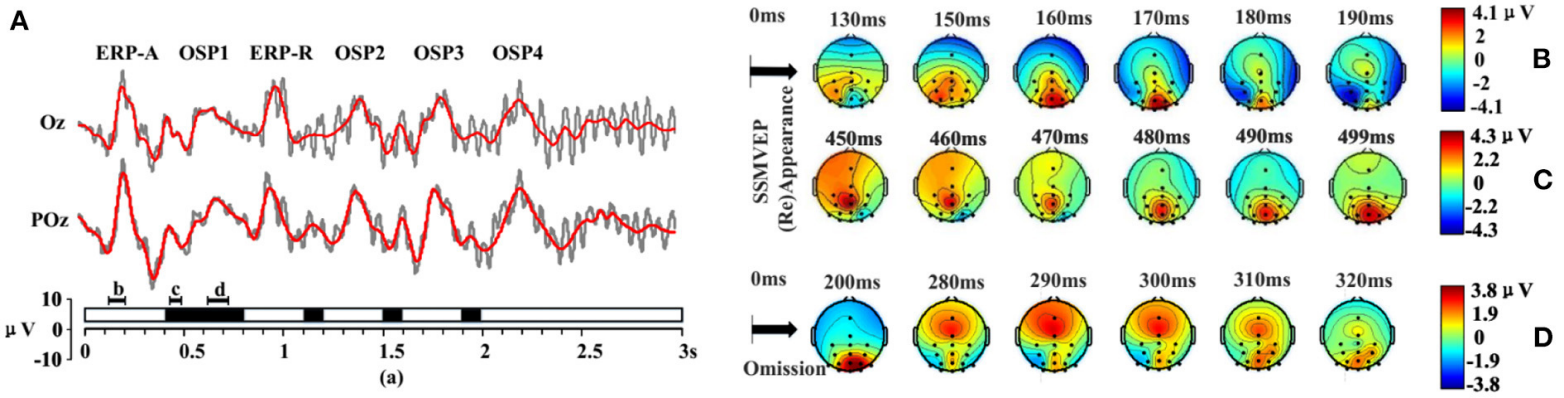
The ERP and OSP feature in the time domain and spatial distribution (64 trials). **(A)** The signal reconstructed based on the EMD method. **(B,C)** The representative scalp topographies of ERP features. **(D)** The representative scalp topographies of OSP feature. The corresponding time of the scalp topographies is illustrated in **(A)**.

Figure 9A shows the reconstructed ERP and OSP signal by adopting the EMD analysis. After filtering out unrelated SSVEP features and noise from the raw signal, the OSP and ERP features were clear with typical peaks. Consistent with Supplementary Figure S5 (see Supporting Document), four OSP features were elicited at about 300 ms after the omitting events. Two features of ERP were found after the onset of Newton's ring at the beginning of the stimuli and the end of long omitting events, with prominent peaks at about 180 and 480 ms after the events. Past research shows that multi-flash evoked in succession may decrease the ERP performance (Jin et al., 2012). However, this phenomenon was not found in our multi-OSP paradigm, in which the peaks of OSP features were almost the same.

Scalp topographies were used to investigate the spatial features of the brain's response to the hybrid paradigm. Series containing representative OSP and ERP features were analyzed (Figures 9B–D). ERP can be found in 500 ms after the appearance or reappearance of SSMVEP, where there are two positive peaks at 130–190 and 450–500 ms. ERP feature of the first peak was distributed in an occipital and parietal region including Oz, O2, PO3, POz, PO4, and Pz, and the second peak was distributed in the parietal and adjacent central and occipital regions (Pz, CPz, and POz). OSP was found in 290–310 ms after the onset of stimulus absence and it seems stronger over frontal, central, parietal, and partly occipital regions (Fz, Cz, Pz, P4, POz, PO4, Oz, and O2). However, the feature at 200 and 320 ms was SSMVEP robustly observed in the occipital region.

b. SSVEP feature.

**Figure 10:**
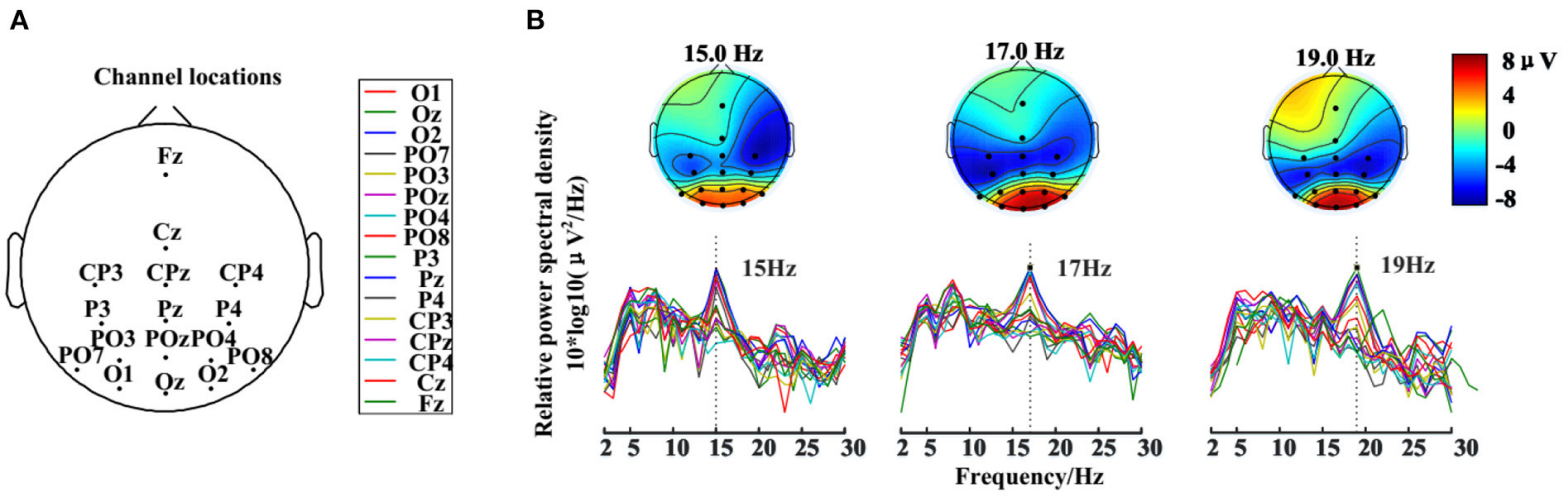
The spatial distribution and power spectral density (PSD) spectrum of the SSVEP feature. **(A)** 16-electrode locations were selected in the experiment. **(B)** PSD spectrum of 16 channels and its corresponding scalp topographies in different stimulus frequencies.

According to the EEGLAB toolbox in MATLAB, Figure 10 gives the power spectral density (PSD) and the corresponding scalp topographies. The SSVEP feature frequencies are still apparent with multi omitting events inserted in the paradigm, as shown in Figure 10B. Different omission sequences did not influence the prominent frequency peaks for the targets with the same stimulation frequency (see Supporting Document: Supplementary Figure S6). After the 6–30 Hz band-pass filter (4th order zero-phase-shift Butterworth filter), SSVEP features were prominent in the visual cortical areas in the occipital and adjacent parietal regions, especially at O1, Oz, O2, POz, PO4, and PO8.

c. Time-Frequency feature.

**Figure 11:**
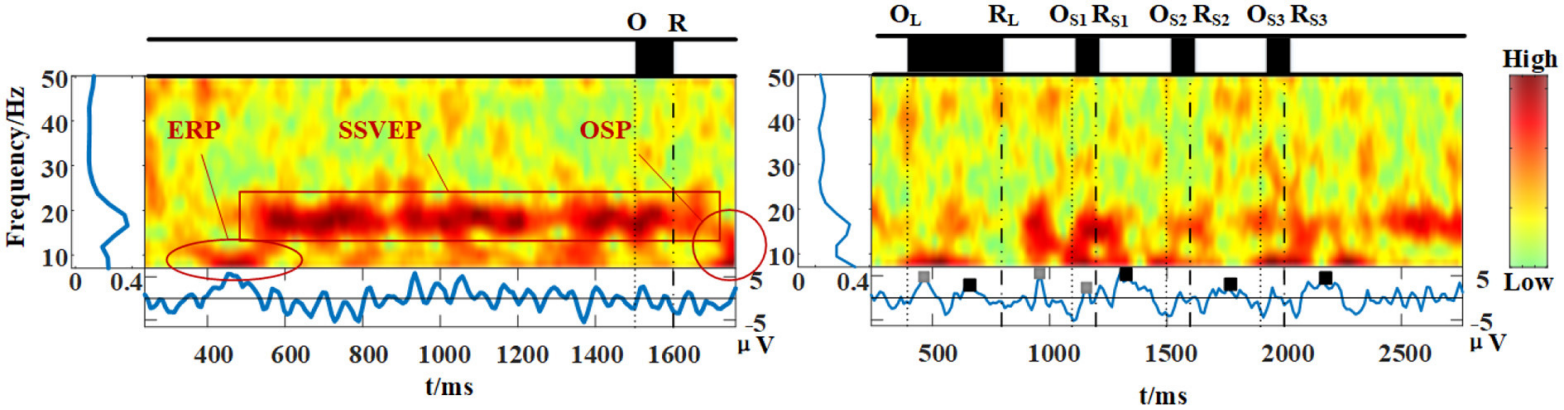
Time-frequency analysis at POz for left: one-time omission and for right: four-times omission including one long omission and three short omissions (64 trials). The stimulation frequency is 15 Hz.

Since this paradigm is a time-frequent joint coding method, it was essential to analyze the features elicited in the time and frequency domains simultaneously. According to the above analysis, a time-frequency analysis based on the represented electrode Poz (presented in Figure 11), as all related features could be found on this site. Like previous studies, SSVEP features were stable before the omitting event and blocking during the event (Xu et al., 2013). However, when multi omitting events were inserted, the SSVEP feature was broken into discontinuous pieces, demonstrating that omission times impacted the stimulus series. Although the frequency of the stimuli was still recognizable in the frequency domain, as shown in the FFT curve, the features of SSVEP were influenced by OSP and ERP when considering the time domain.

The OSP and ERP features in the time-frequency domain were nearly accordant with the above time-domain analysis, with accordant latency. However, they were also influenced by the low-frequency component of SSVEP features, in which the boundary of SSVEP and ERP or OSP was not that clear. This phenomenon was interesting. It might have been caused when the SSVEP stimuli were broken into small fragments by the insertion of multiple OSP, as the time was too short for the SSVEP to evolve from transient to steady-state. This result showed that, as multi-time and frequency information is used together to code the paradigm, it is unavoidable that these features would be influenced by each other.

#### Online performance

**Figure 12:**
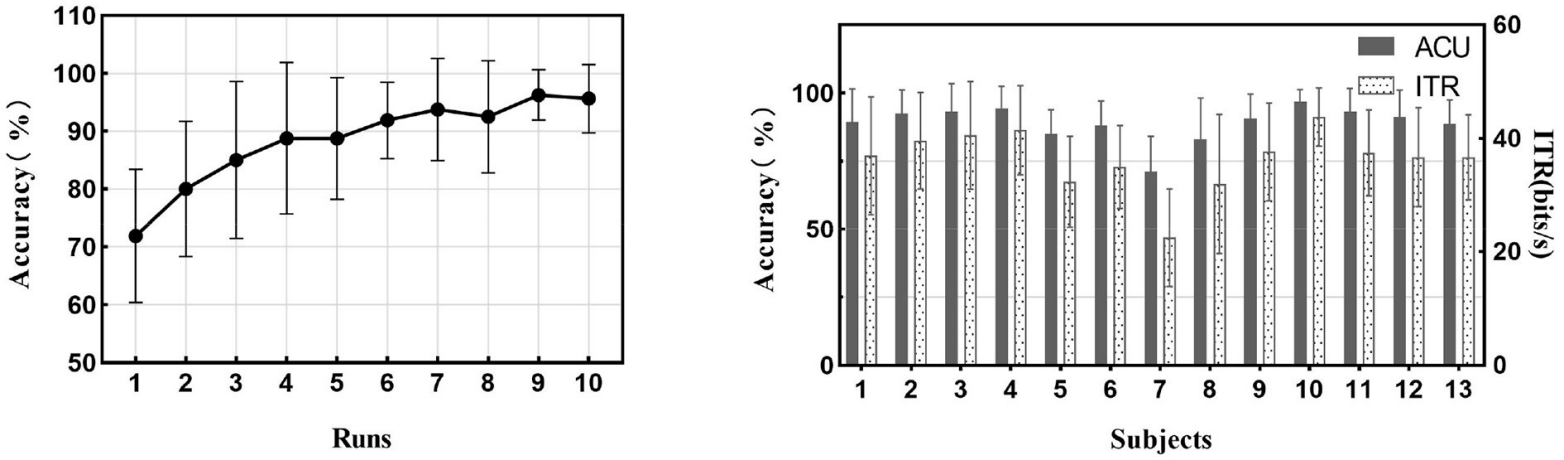
The BCI performance of 13 subjects on the nine-target online experiment. The left figure shows the increasing trend of average accuracies against the number of runs. The figure on the right shows the average performance of trials of each of the 13 subjects.

All the subjects finished the online task, and no unfavorable conditions occurred during the task. The mean accuracy and standard deviation for thirteen subjects were 89.04 ± 6.53%, and the ITR (mean ± SD) for all subjects was 36.37 ± 5.34 bits/min. The accuracy and ITR of all runs for each subject are displayed in Figure 12. The average accuracy for all subjects increased as the runs increased.

## Discussion

### The SSMVEP-OSP paradigm and influencing factors

Previous studies have shown that OSP features could be elicited by omission events in periodic flickering stimuli (Bullock et al., 1994; McCullagh et al., 2009). In our study too, we found this feature to be compatible with the SSMVEP paradigm, showing the same OSP with a positive peak at about 300 ms after the omission (Bullock et al., 1994; McCullagh et al., 2009; Xu et al., 2013, 2014; Wu et al., 2016). This result extends the application range of OSP, without the limitation of the monitor's refresh rate in the periodic flickering stimuli. As presented in Supplementary Figures S3, S4, OSP features seem obvious in nearly all frequencies from 6 to 30 Hz with the step of 1 Hz. In addition, the frequency feature in the spectrogram remained recognizable among most of these frequencies in our experiments. Thus, except for simultaneous eliciting of f-VEP and t-VEP features, more frequency information was available for coding targets in this paradigm compared to existing studies (Xu et al., 2014; Wu et al., 2016). It should be noted that the OSP might have the potential for more extensive application in other prevailing stimuli such as SSVEP based on luminance, given its similarity to SSMVEP (Chen et al., 2014a,b).

The duration of omission is another key factor in this hybrid paradigm. Only one existing study explored the influence of omission duration. Wu et al. (2016) concluded that the omission duration of minimum value (1/Stimulus Frequency) would be better, after briefly comparing it with the duration of 0.1 s. However, after comparing multiple duration, we found that too short or too long durations were both limited. A too short duration might be easily ignored visually and cause a less prominent OSP feature, as shown in Figure 5, while a too long duration might weaken the SSVEP feature and make the duration of the whole stimuli longer. Thus, in our study, we choose a short duration of 0.1s for trade-offs. It is worth noting that the duration of omission would reduce the SSVEP response, as shown in Figures 5, 11. However, this could be resolved by adopting omission with appropriate duration and recognizing both t-VEP and f-VEP features.

When comparing short and long durations of omission, we found an interesting phenomenon that an extra ERP feature was elicited after the reappearance of SSMVEP stimuli, when the omission duration was longer than 400 ms. As far as we know, none of the previous studies have explored how the SSVEP is influenced by preceding omission events in the VEP-BCI paradigm. We surmised that the brain's dynamic response resulted in this phenomenon. This ERP feature was almost the same as the ERP after the SSVEP onset (with the same positive peak at about 180 ms and a similar waveform), consistent with the dynamic response of SSVEP in the previous study (Ferrari et al., 2010). One explanation is that the OSP and ERP interplay with each other (Xu et al., 2016a). In past research, multi-ERP events in succession lead to epoch “overlaps” for P300 features (Martens et al., 2009). While the OSP feature lasts for more than 300 ms, the following ERP may be negatively influenced by similar “overlaps” when OSP and the ERP event are close to 100 ms. Another possible explanation is that, when the omission duration is long, the brain may have enough time to prepare for the subsequent ERP events, similar to the “repetition blindness” effect in the P300 response (Kanwisher, 1987; Salvaris and Sepulveda, 2009).

Combining multi OSPs to evoke multiple t-VEP features in the SSVEP BCI paradigm has not been studied thus far. We verified the feasibility of this method in our study. Moreover, we found that OSPs were not influenced by the interval of events, as the other ERPs (like P300 in oddball) were. In our study of the multi omission paradigm, the peaks of OSP features were not attenuated when arranged with an interval of 400 ms (Figures 7, 9). Previous studies have shown that the evoked P300 response would be attenuated when two identical targets were presented in a stream of non-targets at intervals of <500 ms influenced by the “repetition blindness” phenomenon (Kanwisher, 1987; Salvaris and Sepulveda, 2009; Jin et al., 2011). However, the ERP with background SSVEP was less influenced by this phenomenon. Previous studies have shown that the ERP varies with different background SSVEP, which might be caused by the non-linear phase resetting of neural oscillations (Xu et al., 2014, 2016a,b). Similarly, in our study, the Transient-State Response (TSR, namely ERP here) was reset faster by the Steady-State Response (SSR, namely SSVEP here), to allow shorter intervals for an intact ERP response in the stimuli events that followed. However, further studies are needed to understand this finding.

The disappearance pattern of omissions showed a higher amplitude of OSP compared with the blocking pattern. This finding is similar to a previous study, in which the two patterns were missing white and missing black under the black-white flickering SSVEP paradigm (Wu et al., 2016). It might be explained that, as a P300-like ERP, the OSP would also be modulated by both stimulus significance and novelty (Bullock et al., 1994; Chen et al., 2014b). Compared with the OSP response evoked by the SSVEP stimuli omission in the blocking pattern, an extra ERP evoked by the concurrent disappearing event would be superimposed. Thus, the response reinforced by the disappearing pattern brought more novel and significant visual perception.

### The coding method

**Figure 13:**
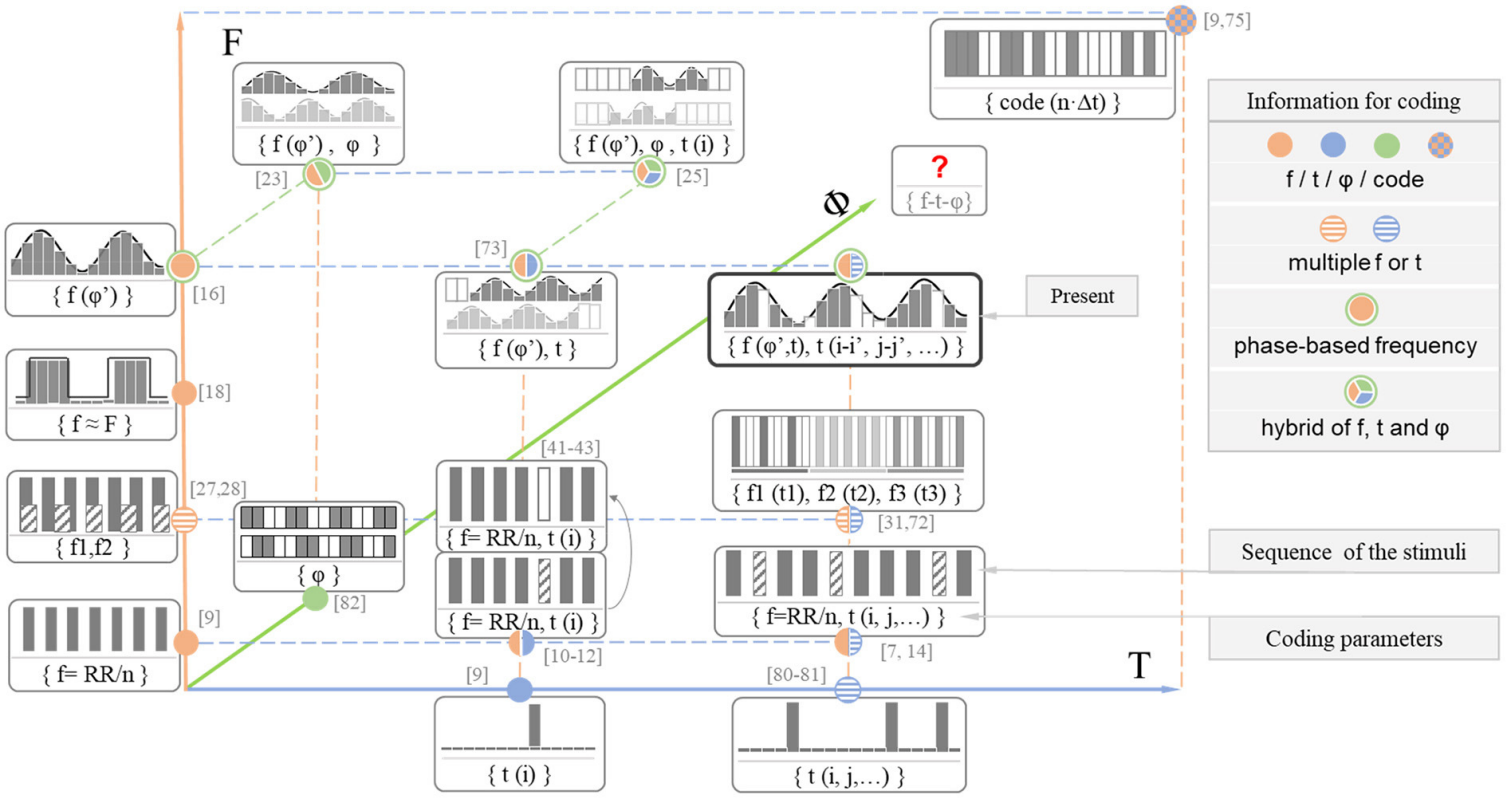
A comparison of coding methods on the current VEP BCI paradigm based on time, frequency, phase, and code information.

The MTFSC is a novel time-frequency coding method proposed in this research that could elicit abundant features, including SSVEP, OSP, ERP, and timing and permutation of SSVEP-OSPs. As a result, this method can use more information from time and frequency domains simultaneously compared to other frequently used VEP coding methods. Figure 13 illustrates the comparison between these VEP-based BCIs from the perspective of the coding method design.

**Figure 14:**
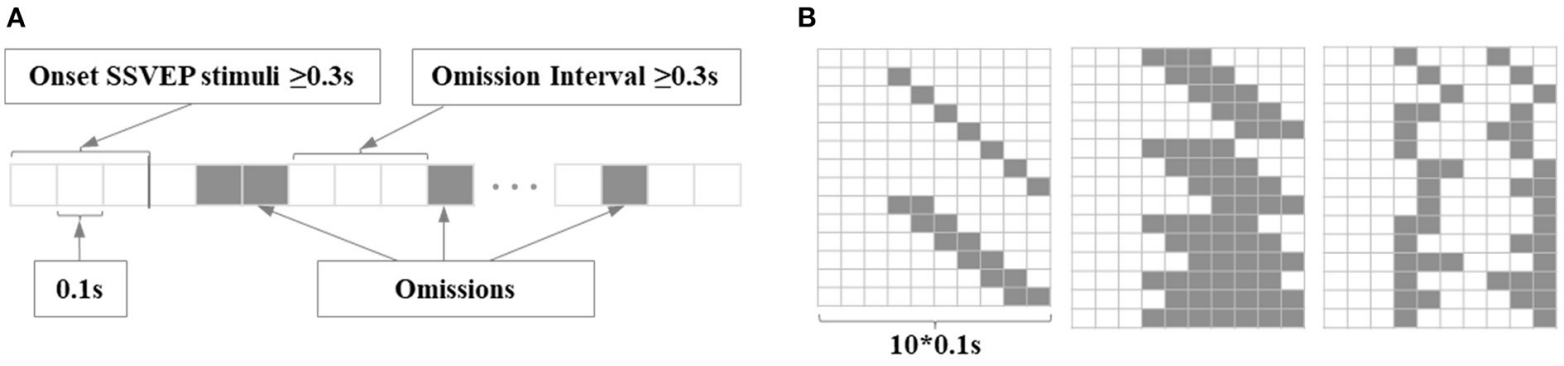
**(A)** An extended application of the MTFSC method. Each square represents the 0.1s stimuli. Omissions with different duration and numbers are inserted into the SSVEP stimuli. The onset of SSVEP stimuli and the interval between omissions must be longer than 0.3 s to produce stable SSVEP and OSP features. **(B)** An example of all stimuli sequences of 1 s stimuli produced by the MTFSC method under one stimulation frequency. Each row represents one specific stimuli sequence, for a total of 44.

Unlike adopting different visual elements such as color and symbol to elicit ERP or SSVEP in the early studies about the time-frequency coding method, our study adopts a visually simple method to elicit multiple time and frequency features only by SSVEP stimuli with omissions (Yin et al., 2013b, 2015; Allison et al., 2014; Wang et al., 2015; Chang et al., 2016). Xu et al. proposed a time-frequency coding strategy in which the interruption of the flicker was similar to the omission in our research (Xu et al., 2014). However, only one omission with a duration of constant 200 ms was used in their method. Besides, they used an extra LED device to provide stimulation frequency, which was not convenient to use on the computer. Using sequential coding methods for time-frequency paradigms has also been studied in previous research (Zhang et al., 2012; Kimura et al., 2013; Dehzangi et al., 2014). However, only frequency sequences (sequence with multi-frequency) were used in their research. The information per unit stimulation time was limited because each epoch only offered one frequency information, while time information was not used. To use the time information in SSVEP stimuli, Lin et al. (2016) tried to use SSVEP with two different onsets (0 or 0.5 s) to discriminate the SSVEP paradigm. This method was similar to the SSVEP combined with a single OSP (occur in different moments) and improved the ITR and accuracy in their study. Similarly, Xu et al. adopted the same method but used shorter SSVEP stimuli (200 ms) with nine different onsets (0–0.8 s with the step of 0.1 s), and generated nine sub-targets in a single SSVEP stimulus (Xu et al., 2020). Although only three sequences were demonstrated in our study, the MTFSC method has great potential in coding far more targets in a single SSVEP stimulus. Fig.13 demonstrates an extended application of the MTFSC method. The 1 s stimulus could generate 44 sequences in this method under a specific frequency of the stimulus, as shown in Figure 14. Table 1 lists the number of stimuli sequences generated theoretically in the different stimuli durations and different omission numbers under the extended MTFSC protocol. Compared with previous research, a slight increase in the stimuli duration in this method largely improved the number of targets (Zhang et al., 2012; Dehzangi et al., 2014; Xu et al., 2020).

**Table 1:** The number of stimuli sequences that can be generated in the different stimuli duration when different omission numbers are used under the extended MTFSC protocol, with only one stimulus frequency used.

**Stimuli duration**	**Omission number**						**Total number**
	**0**	**1**	**2**	**3**	**…**	***N***	
8*0.1 S	1	15	1	/	/	/	17
10*0.1 s	1	28	15	/	/	/	44
12*0.1 s	1	45	70	1	/	/	117
…	1	…	…	…	…	…	…
M*0.1 s	1	$\sum_{\text{k}=1}^{\text{M}-3}k$	$\sum_{\text{k}=1}^{M-7}[(M-6-k)\;*\sum_{j=1}^{k}j]$	$\sum_{\text{k}=1}^{M-11}[(M-10-k)\;*\sum_{j=1}^{k}j]$	…	$\sum_{\text{k}=1}^{M-4\text{N}+1}[(M-4N+2-k)\;*\sum_{j=1}^{k}j]$	…

Other recent studies have combined the phase and frequency information for coding hybrid paradigms (Manyakov et al., 2012; Chen et al., 2015; Xu et al., 2020). Although we used the motion phase in our method, it was only used to break through the limitation of the monitor's refresh rate on SSVEP stimuli, instead of coding extra information as these studies do. Notably, our MTFSC is compatible with the phase-frequency joint coding method because previous studies had integrated the phase and frequency into an SSVEP stimulus of 0.2 s duration, while the SSVEP stimuli fragment in our study was longer than 0.3 s.

We also compared these methods with the c-VEP method, similar to the t-VEP and f-VEP methods. The c-VEP method fully used the time information in every frame to code the targets. Although the features in the single frame are blurred, the coding feature formed by these features helps to identify the specific target. It was interesting to note that, visually speaking (Figure 13), these stimuli look more like the c-VEP stimuli as more f-VEP information was used in f-VEP stimuli, compared to those adopting only one periodic code or event (Mukesh et al., 2005; Zhang et al., 2012; Hwang et al., 2013; Yin et al., 2013a, 2015; Dehzangi et al., 2014). Besides, the target recognition method was also similar. The template matching method had been well used in the c-VEP. Currently, this method is increasingly used in recent time-frequency coding method research with good performance (Mukesh et al., 2005; Jia et al., 2010; Zhang et al., 2012; Hwang et al., 2013; Chen et al., 2015, 2017; Nakanishi et al., 2015; Xu et al., 2020). Perhaps the method using the template containing more coupled time-frequency information performs better than separately using simple frequency or time characteristics when recognizing the coupling characteristics coded by more time-frequency information. As shown in Figure 11, when multiple time-frequency information was used in coding, the time-frequency graph was more blurred compared with the one omission event. Besides, the comparison presented in Supplementary Figure S8 (see Supporting Document) also showed that the accuracy based on the hybrid recognition method was better than the step-by-step method (recognizing SSVEP and ERP separately). Thus, the experience in studying the c-VEP might be used to help further use the time information while being compatible with other frequency and phase information.

### Limitations and direction for future research

Recent research on BCI for communication largely improved performance in ITR (over 200 bits/s) or the number of targets (over 100 targets) (Chen et al., 2015; Nakanishi et al., 2017; Xu et al., 2020). Although our study focused more on the feasibility of the novel coding method instead of pursuing performance improvement, it is still meaningful to compare and analyze the limitation of our study for further improvement.

The 3 s duration of the stimuli cycle in our study is too long, compared with the 1 s stimuli cycle in other research (Chen et al., 2015; Xu et al., 2020). It would lead to severe decreases in ITR, considering that the time duration stimuli are the denominator in the ITR formula. Thus, to improve the ITR in future research, the primary task is to decrease the duration of the stimuli cycle. It is worth mentioning that our MTFSC method increased the information without sacrificing time because the time information was inserted in the SSVEP stimuli. As shown in Figure 14 and Table 1, the MTFSC method code even had the capacity to code more targets under the short stimuli cycle. Another key limitation is that we only used nine targets while other studies used dozens or even over 100 targets. However, increasing the number of targets might largely decrease the accuracy of the paradigm if it is unable to carry enough discriminated information. In fact, we made a preliminary test of 28 targets (7 frequencies and 4 omission sequences) among five subjects. As shown in Supplementary Figure S9, the accuracy was slightly decreased while the ITR was nearly doubled compared with the nine target performance. Thus, there is still room for improving the MTFSC method based paradigm.

To further improve the MTFSC method, we have to do a trade-off between coding more information in a limited time and being recognizable. Studies focused on the brain's dynamic response to VEP are essential for guiding this trade-off, considering that all visual stimuli would be filtered and interplay with each other in the non-linear neural pathway. For instance, in c-VEP, the autocorrelation function of the evoked potential would not keep the same sharpness as in the visual stimuli sequence (Bin et al., 2011; Wei et al., 2018). In addition, as previous studies have shown, the ERP varies with different background SSVEP. In our study, the “blind repetition” phenomenon in the pure ERP paradigm was not obvious when combined with the SSVEP paradigm (Kanwisher, 1987; Salvaris and Sepulveda, 2009; Jin et al., 2011). These findings on the EEG dynamic response show that SSVEP stimuli and omission events reduced the negative impact of conventional ERP stimuli. Thus, it would help design a better paradigm, such as the extended MTFSC method protocol, by fully using time-frequency information. Recent studies that focus on exploring dynamic responses (Xu et al., 2016a; Zhang et al., 2018) contribute to other research to improve performance, such as shortening the stimuli duration and training time (Han et al., 2019; Xu et al., 2020). In the recent research by Xu et al., by referencing the study of a dynamic model, an SSVEP and ERP stimuli as short as 200 ms were proposed along with a significant expansion in the number of targets (Xu et al., 2014, 2016a, 2020).

These dynamic responses help us understand how the brain works (Xu et al., 2016a; Zhang et al., 2018). For example, Xu. et al. studied the dynamic model of ERPs under the SSVEP pre-stimulus and revealed the “three-period-transition” for ERP generation (Xu et al., 2016a). However, our study provides just a brief presentation of the dynamic response while further experiments are needed to study the exact mechanism of the dynamic response as in these studies. Thus, more quantitative studies on the dynamic response of our hybrid paradigm are required. The MTFSC method serves as a perfect tool to research the dynamic response of the brain to VEP stimuli. Because multiple f-VEP and t-VEP stimuli fragments are used together, many SSR and TSR are involved and interplayed. Therefore, many factors could be included to study the dynamic model conveniently for each fragment. For example, when considering each stimuli fragment's phase, we could study whether the stimuli that follow in a consistent phase would strengthen the SSR or if the stimuli that follow with the antiphase would rapidly attenuate the TSR as in other methods (Notbohm et al., 2016; Zhang et al., 2018; Otero et al., 2020). These studies would help us optimize the time-frequency paradigm and evaluate whether extra phase information could be coded together with this MTFSC method.

Overall, the MSTFC method shows great potential in building a BCI communication system with a large number of command codes. The proposed method would promote the application in scenes that demand large control orders, for example, the spelling keyboard that supports multiple languages (like English and Chinese), or the GUI controlling an operation system developed for assisting paralyzed patients in a practical environment. Besides, the extended MSTFC method shows a great capacity to contain more time information while being compatible with other VEP-BCI systems. Further studies that shorten the stimuli cycle in this study would also be helpful for people who need to do complex tasks on multiple devices in a concerted manner.

## Conclusion

This study proved that the SSMVEP and multi-OSP could be used in a hybrid paradigm to produce robust and abundant time and frequency features. OSP features seem obvious in nearly all frequencies, from 6 to 30 Hz with the step of 1 Hz. We found an interesting phenomenon of an extra ERP feature being elicited after the reappearance of SSMVEP stimuli when omission duration was longer than 400 ms. This study also proposed a novel MTFSC coding method, which was investigated according to the preliminary results. Time information could be incorporated into frequency modulated BCI. Although these time and frequency features deeply interplay with each other according to the time-frequency spectrum analysis, the feature is still recognizable with the template-based CCA method. The MTFSC is feasible and efficient, eliciting time and frequency features including the multi-OSP, ERP, and SSVEP. The preliminary online analysis showed that the accuracy and the ITR (mean ± standard deviation) of a nine-target stimulator with 13 subjects were 89.04% and 36.37 bits/min. The MTFSC proved feasible and shows great potential in improving performance by better using time information in specific stimulation frequencies. This study holds promise for developing better BCI systems with a novel coding method.

## Data availability statement

The raw data supporting the conclusions of this article will be made available by the authors, without undue reservation.

## Ethics statement

The studies involving human participants were reviewed and approved by the Institutional Review Board of Xi'an Jiaotong University. The patients/participants provided their written informed consent to participate in this study.

## Author contributions

ZY designed the algorithm applied in this paper and conducted the experiment. YP and JW provided consultants and reviewed this paper. BS helped with the experiment. QW, S-YR, and ML helped in polishing the paper. All authors contributed to the article and approved the submitted version.

## Funding

This work was funded by Henan Province Medical Science and Technology Research Program (SBGJ202002092).

## Conflict of interest

The authors declare that the research was conducted in the absence of any commercial or financial relationships that could be construed as a potential conflict of interest.

## Publisher's note

All claims expressed in this article are solely those of the authors and do not necessarily represent those of their affiliated organizations, or those of the publisher, the editors and the reviewers. Any product that may be evaluated in this article, or claim that may be made by its manufacturer, is not guaranteed or endorsed by the publisher.

## References

[B1] Allison Z. Jin B. Zhang J. Y. Wang X. (2014). A four-choice hybrid P300/SSVEP BCI for improved accuracy. Brain-Comput. Int. 1, 17–26. 10.1080/2326263X.2013.869003

[B2] Bin G. Gao X. Wang Y. Hong B. Gao S. (2009a). VEP-based brain-computer interfaces: time, frequency, and code modulations. IEEE Comput. Intell. Mag. 4, 22–26. 10.1109/MCI.2009.934562

[B3] Bin G. Gao X. Wang Y. Li Y. Hong B. Gao S.. (2011). A high-speed BCI based on code modulation VEP. J. Neural Eng. 8, 025015. 10.1088/1741-2560/8/2/025015 21436527

[B4] Bin G. Gao X. Yan Z. Hong B. Gao S. (2009b). An online multi-channel SSVEP-based brain–computer interface using a canonical correlation analysis method. J. Neural Eng., 6, 046002. 10.1088/1741-2560/6/4/046002 19494422

[B5] Bullock T. H. Karamürsel S. Achimowicz J. Z. McClune M. C. Başar-Eroglu C. (1994). Dynamic properties of Hum. visual evoked and omitted stimulus potentials. Electroencephalogr. Clin. Neurophysiol. 91, 42–53. 10.1016/0013-4694(94)90017-5 7517843

[B6] Chai X. Zhang Z. Guan K. Liu G. Niu H. (2019). A radial zoom motion-based paradigm for steady state motion visual evoked potentials. Front. Hum. Neurosci. 13, p.127. 10.3389/fnhum.2019.00127 31040775 PMC6477057

[B7] Chang M. H. Baek H. J. Lee S. M. Park K. S. (2014). An amplitude-modulated visual stimulation for reducing eye fatigue in SSVEP-based brain-computer interfaces. Clin. Neurophysiol. 125, 1380–1391. 10.1016/j.clinph.2013.11.016 24368034

[B8] Chang M. H. Lee J. S. Heo J. Park K. S. (2016). Eliciting dual-frequency SSVEP using a hybrid SSVEP-P300 BCI. J. Neurosci. Meth. 258, 104–113. 10.1016/j.jneumeth.2015.11.001 26561770

[B9] Chaudhary U. Birbaumer N. Ramos-Murguialday A. (2016). Brain-computer interfaces for communication and rehabilitation. Nat. Rev. Neurol. 12, 513. 10.1038/nrneurol.2016.113 27539560

[B10] Chen S. J. Peng C. J. Chen Y. C. Hwang Y. R. Lai Y. S. Fan S. Z.. (2016). Comparison of FFT and marginal spectra of EEG using empirical mode decomposition to monitor anesthesia. Comput. Math. Prog. Biomed. 137, 77–85. 10.1016/j.cmpb.2016.08.024 28110742

[B11] Chen X. Chen Z. Gao S. Gao X. (2014a). A high-ITR SSVEP-based BCI speller. Brain-Comput. Interfaces 1, 181–191. 10.1080/2326263X.2014.944469

[B12] Chen X. Wang Y. Nakanishi M. Gao X. Jung T. P. Gao S.. (2015). High-speed spelling with a noninvasive brain–computer interface. Proc. Natl. Acad. Sci. 112, E6058–E6067. 10.1073/pnas.1508080112 26483479 PMC4640776

[B13] Chen X. Wang Y. Nakanishi M. Jung T. P. Gao X. (2014b). Hybrid frequency and phase coding for a high-speed SSVEP-based BCI speller, in 2014 36th Annual Int. Conference of the IEEE Eng in Medicine and Biology Society (Piscataway, NJ: IEEE), 3993–3996. 10.1109/EMBC.2014.6944499 25570867

[B14] Chen X. Wang Y. Zhang S. Gao S. Hu Y. Gao X.. (2017). A novel stimulation method for multi-class SSVEP-BCI using intermodulation frequencies. J. Neural Eng., 14, 026013. 10.1088/1741-2552/aa5989 28091397

[B15] Chen Y. Bayesteh A. Wu Y. Ren B. Kang S. Sun S.. (2018). Toward the standardization of non-orthogonal multiple access for next generation wireless networks. IEEE Commun.Mag. 56, 19–27. 10.1109/MCOM.2018.1700845

[B16] Choi I. Rhiu I. Lee Y. Yun M. H. Nam C. S. (2017). A systematic review of hybrid brain-computer interfaces: taxonomy and usability perspectives. PloS ONE, 12, e0176674. 10.1371/journal.pone.0176674 28453547 PMC5409179

[B17] Combaz A. Van Hulle M. M. (2015). Simultaneous detection of P300 and steady-state visually evoked potentials for hybrid brain-computer interface. PloS ONE, 10, e0121481. 10.1371/journal.pone.0121481 25815815 PMC4376875

[B18] David O. Kilner J. M. Friston K. J. (2006). Mechanisms of evoked and induced responses in MEG/EEG. Neuroimage 31, 1580–1591. 10.1016/j.neuroimage.2006.02.034 16632378

[B19] Dawson G. D. (1954). A summation technique for the detection of small evoked potentials. Electroencephalogr. Clin. Neurophysiol. 6, 65–84 10.1016/0013-4694(54)90007-3 13141922

[B20] De Neeling M. Van Hulle M. M. (2019). Single-paradigm and hybrid brain computing interfaces and their use by disabled patients. J. Neural Eng. 16, 061001. 10.1088/1741-2552/ab2706 31163412

[B21] Dehzangi O. Nathan V. Zong C. Lee C. Kim I. Jafari R.. (2014). A novel stimulation for multi-class SSVEP-based brain-computer interface using patterns of time-varying frequencies, in 2014 36th Annual Int. Conference of the IEEE Eng. in Medicine and Biology Society (https://www.google.com/search?q=Piscataway&stick=H4sIAAAAAAAAAOPgE-LQz9U3SCvIylICsyoKCou0tLKTrfTzi9IT8zKrEksy8&sa=X&ved=2ahUKEwjD0aTR3fX4AhU_fzABHatzC9QQmxMoAHoFCIgBEAI Piscataway, NJ: IEEE), 118–121 10.1109/EMBC.2014.6943543 25569911

[B22] Donchin E. Spencer K. M. Wijesinghe R. (2000). The mental prosthesis: assessing the speed of a P300-based brain-computer interface. IEEE Transac. Rehabil. Eng., 8, 174–179. 10.1109/86.847808 10896179

[B23] Edlinger G. Guger C. (2012). A hybrid brain-computer interface for improving the usability of a smart home control, in 2012 ICME Int. Conference on Complex Medical Eng. (CME) (Piscataway, NJ: IEEE), 182–185.

[B24] Fan X. A. Bi L. Teng T. Ding H. Liu Y. (2014). A brain–computer interface-based vehicle destination selection system using P300 and SSVEP signals. IEEE Transac. Intell. Transport. Syst. 16, 274–283. 10.1109/TITS.2014.2330000

[B25] Farwell L. A. Donchin E. (1988). Talking off the top of your head: toward a mental prosthesis utilizing event-related brain potentials. Electroencephalogr. Clin. Neurophysiol. 70, 510–523. 10.1016/0013-4694(88)90149-6 2461285

[B26] Ferrari V. Bradley M. M. Codispoti M. Lang P. J. (2010). Detecting novelty and significance. J. Cogn. Neurosci. 22, 404–411. 10.1162/jocn.2009.21244 19400680 PMC3612945

[B27] Flandrin P. Rilling G. Goncalves P. (2004). Empirical mode decomposition as a filter bank. IEEE Signal Process. Lett. 11, 112–114. 10.1109/LSP.2003.821662

[B28] Han X. Zhang S. Gao X. (2019). A study on reducing training time of BCI system based on an SSVEP dynamic model, in 2019 7th Int. Winter Conference on Brain-Computer Interface (BCI) (https://www.google.com/search?q=Piscataway&stick=H4sIAAAAAAAAAOPgE-LQz9U3SCvIylICsyoKCou0tLKTrfTzi9IT8zKrEksy8XAAAA&sa=X&ved=2ahUKEwjD0aTR3fX4AhU_fzABHatzC9QQmxMoAHoFCIgBEAI Piscataway, NJ: IEEE), 1–2.

[B29] Herrmann C. S. (2001). Hum. EEG responses to 1–100 Hz flicker: resonance phenomena in visual cortex and their potential correlation to cognitive phenomena. Exp. Brain Res. 137, 346–353. 10.1007/s002210100682 11355381

[B30] Hotelling H. (1992). Relations between two sets of variates, in Breakthroughs in statistics (New York, NY: Springer), 162–190.

[B31] Hwang H. J. Kim D. H. Han C. H. Im C. H. (2013). A new dual-frequency stimulation method to increase the number of visual stimuli for multi-class SSVEP-based brain–computer interface (BCI). Brain Res. 1515, 66–77. 10.1016/j.brainres.2013.03.050 23587933

[B32] Jia C. Gao X. Hong B. Gao S. (2010). Frequency and phase mixed coding in SSVEP-based brain-computer interface. IEEE Transac. Biomed. Eng. 58, 200–206. 10.1109/TBME.2010.2068571 20729160

[B33] Jin J. Allison B. Z. Sellers E. W. Brunner C. Horki P. Wang X.. (2011). An adaptive P300-based control system. J. Neural Eng. 8, 036006. 10.1088/1741-2560/8/3/036006 21474877 PMC4429775

[B34] Jin J. Sellers E. W. Wang X. (2012). Targeting an efficient target-to-target interval for P300 speller brain–computer interfaces. Med. Biol. Eng. Comput. 50, 289–296. 10.1007/s11517-012-0868-x 22350331 PMC3646326

[B35] Kanwisher N. G. (1987). Repetition blindness: Type recognition without token individuation. Cognition 27, 117–143. 10.1016/0010-0277(87)90016-3 3691023

[B36] Katyal A. Singla R. (2020). A novel hybrid paradigm based on steady state visually evoked potential and P300 to enhance information transfer rate. Biomed. Signal Process. Control 59, 101884. 10.1016/j.bspc.2020.101884

[B37] Kerr B. (1973). Processing demands during mental operations. Mem. Cogn. 1, 401–412. 10.3758/BF03208899 24214632

[B38] Kimura Y. Tanaka T. Higashi H. Morikawa N. (2013). SSVEP-based brain-computer interfaces using FSK-modulated visual stimuli. IEEE Transac. Biomed. Eng. 60, 2831–2838. 10.1109/TBME.2013.2265260 23739780

[B39] Krusienski D. J. Sellers E. W. Cabestaing F. Bayoudh S. McFarland D. J. Vaughan T. M.. (2006). A comparison of classification techniques for the P300 Speller. J. Neural Eng. 3, 299. 10.1088/1741-2560/3/4/007 17124334

[B40] Leamy D. J. Ward T. E. (2010). A novel co-locational and concurrent fNIRS/EEG measurement system: design and initial results, in 2010 Annual Int. Conference of the IEEE Eng. in Medicine and Biology (New York, NY: IEEE), 4230–4233 10.1109/IEMBS.2010.5627377 21096900

[B41] Lin K. Wang Y. Gao X. (2016). Time-frequency joint coding method for boosting information transfer rate in an SSVEP based BCI system, in 2016 38th Annual Int. Conference of the IEEE Eng. in Medicine and Biology Society (EMBC) (New York, NY: IEEE), 5873–5876. 10.1109/EMBC.2016.7592064 28269590

[B42] Lin Z. Zhang C. Wu W. Gao X. (2006). Frequency recognition based on canonical correlation analysis for SSVEP-based BCIs. IEEE Transac. Biomed. Eng. 53, 2610–2614. 10.1109/TBME.2006.886577 17152442

[B43] Liu P. Li Z. (2012). Task complexity: A review and conceptualization framework. Int. J. Indus. Ergon. 42, 553–568. 10.1016/j.ergon.2012.09.001

[B44] Manyakov N. V. Chumerin N. Van Hulle M. M. (2012). Multichannel decoding for phase-coded SSVEP brain–computer interface. Int. J. Neural Syst. 22, 1250022. 10.1142/S0129065712500220 22963395

[B45] Martens S. M. M. Hill N. J. Farquhar J. Schölkopf B. (2009). Overlap and refractory effects in a brain–computer interface speller based on the visual P300 event-related potential. J. Neural Eng. 6, 026003. 10.1088/1741-2560/6/2/026003 19255462

[B46] McCullagh J. Weihing J. Musiek F. (2009). Comparisons of P300s from standard oddball and omitted paradigms: implications to exogenous/endogenous contributions. J. Am. Acad. Audiol. 20, 187–95. 10.3766/jaaa.20.3.5 19927689

[B47] Molina G. G. (2007). BCI adaptation using incremental-SVM learning, in 2007 3rd Int. IEEE/EMBS Conference on Neural Eng. (New York, NY: IEEE), 337–341.

[B48] Moratti S. Clementz B. A. Gao Y. Ortiz T. Keil A. (2007). Neural mechanisms of evoked oscillations: stability and interaction with transient events. Hum. Brain Mapp. 28, 1318–1333. 10.1002/hbm.20342 17274017 PMC6871406

[B49] Mukesh T. S. Jaganathan V. Reddy M. R. (2005). A novel multiple frequency stimulation method for steady state VEP based brain computer interfaces. Physiological Measurement, 27, 61. 10.1088/0967-3334/27/1/006 16365511

[B50] Nakanishi M. Wang Y. Chen X. Wang Y. T. Gao X. Jung T. P.. (2017). Enhancing detection of SSVEPs for a high-speed brain speller using task-related component analysis. IEEE Transac. on Biomed. Eng., 65, 104–112. 10.1109/TBME.2017.2694818 28436836 PMC5783827

[B51] Nakanishi M. Wang Y. Wang Y. T. Jung T. P. (2015). A comparison study of canonical correlation analysis based methods for detecting steady-state visual evoked potentials. PloS ONE, 10, e0140703. 10.1371/journal.pone.0140703 26479067 PMC4610694

[B52] Notbohm A. Kurths J. Herrmann C. S. (2016). Modification of brain oscillations via rhythmic light stimulation provides evidence for entrainment but not for superposition of event-related responses. Front. Hum. Neurosci., 10, 10. 10.3389/fnhum.2016.00010 26869898 PMC4737907

[B53] Otero M. Prado-Gutiérrez P. Weinstein A. Escobar M. J. El-Deredy W. (2020). Persistence of eeg alpha entrainment depends on stimulus phase at offset. Front. Hum. Neurosci. 14, p.139. 10.3389/fnhum.2020.00139 32327989 PMC7161378

[B54] Pfurtscheller G. Allison B. Z. Bauernfeind G. Brunner C. Solis Escalante T. Scherer R.. (2010). The hybrid BCI. Front. Neurosci. 4, 3. 10.3389/fnpro.2010.00003 20582271 PMC2891647

[B55] Salvaris M. Sepulveda F. (2009). Perceptual errors in the Farwell and Donchin matrix speller, in 2009 4th Int. IEEE/EMBS Conference on Neural Eng (New York, NY: IEEE), 275–278.

[B56] Schwent V. L. Hillyard S. A. Galambos R. (1976). Selective attention and the auditory vertex potential. II. Effects of signal intensity and masking noise. Electroencephalogr. Clin. Neurophysiol. 40, 615–622. 10.1016/0013-4694(76)90136-X 57047

[B57] Singla R. Jatana S. (2017). The types of hybrid modalities in brain-computer interface systems: a review. Int. J. Biomed. Eng. Technol. 23, 48–58. 10.1504/IJBET.2017.08222833568979

[B58] Squires K. C. Donchin E. Herning R. I. McCarthy G. (1977). On the influence of task relevance and stimulus probability on event-related-potential components. Electroencephalogr. Clin. Neurophysiol. 42, 1–14. 10.1016/0013-4694(77)90146-8 64341

[B59] Stawicki P. Volosyak I. (2020). Comparison of modern highly interactive flicker-free steady state motion visual evoked potentials for practical brain–computer interfaces. Brain Sci. 10, 686. 10.3390/brainsci10100686 32998379 PMC7601073

[B60] Sutter E. E. (1992). The brain response interface: communication through visually-induced electrical brain responses. J. Microcomput. Appl. 15, 31–45. 10.1016/0745-7138(92)90045-7

[B61] Tsoneva T. Garcia-Molina G. Desain P. (2015). Neural dynamics during repetitive visual stimulation. J. Neural Eng. 12, 066017. 10.1088/1741-2560/12/6/066017 26479469

[B62] Volosyak I. Cecotti H. Gräser A. (2009). Impact of frequency selection on LCD screens for SSVEP based brain-computer interfaces, in Int. Work-Conference on Artificial Neural Networks (Berlin, Heidelberg: Springer), 706–713.

[B63] Wang M. Daly I. Allison B. Z. Jin J. Zhang Y. Chen L.. (2015). A new hybrid BCI paradigm based on P300 and SSVEP. J. Neurosci. Meth. 244, 16–25. 10.1016/j.jneumeth.2014.06.003 24997343

[B64] Wang Y. Jung T. P. (2010). Visual stimulus design for high-rate SSVEP BCI. Electron. Lett. 46, 1057–1058. 10.1049/el.2010.0923

[B65] Wang Y. Wang R. Gao X. Hong B. Gao S. (2006). A practical VEP-based brain-computer interface. IEEE Transac. Neural Syst. Rehabil. Eng. 14, 234–240. 10.1109/TNSRE.2006.875576 16792302

[B66] Wei Q. Liu Y. Gao X. Wang Y. Yang C. Lu Z.. (2018). A novel c-VEP BCI paradigm for increasing the number of stimulus targets based on grouping modulation with different codes. IEEE Transac. Neural Syst. Rehabil. Eng. 26, 1178–1187. 10.1109/TNSRE.2018.2837501 29877842

[B67] Wolpaw J. R. Birbaumer N. McFarland D. J. Pfurtscheller G. Vaughan T. M. (2002). Brain-computer interfaces for communication and control. Clin. Neurophysiol. 113, 767–791 10.1016/S1388-2457(02)00057-3 12048038

[B68] Wu Y. Li M. Wang J. (2016). Toward a hybrid, brain-computer interface based on repetitive visual stimuli with missing events. J. NeuroEng. Rehabil. 13, 1–12. 10.1186/s12984-016-0179-9 27460070 PMC4962511

[B69] Xie J. Xu G. Wang J. Zhang F. Zhang Y. (2012). Steady-state motion visual evoked potentials produced by oscillating Newton's rings: implications for brain-computer interfaces. PloS ONE 7, e39707. 10.1371/journal.pone.0039707 22724028 PMC3378577

[B70] Xu M. Chen L. Zhang L. Qi H. Ma L. Tang J.. (2014). A visual parallel-BCI speller based on the time-frequency coding strategy. J. Neural Eng. 11, 026014. 10.1088/1741-2560/11/2/026014 24608672

[B71] Xu M. Han J. Wang Y. Jung T. P. Ming D. (2020). Implementing over 100 command codes for a high-speed hybrid brain-computer interface using concurrent P300 and SSVEP features. IEEE Transac. Biomed. Eng. 67, 3073–3082. 10.1109/TBME.2020.2975614 32149621

[B72] Xu M. Jia Y. Qi H. Hu Y. He F. Zhao X.. (2016a). Use of a steady-state baseline to address evoked vs. oscillation models of visual evoked potential origin. Neuroimage 134, 204–212. 10.1016/j.neuroimage.2016.03.073 27039704

[B73] Xu M. Qi H. Wan B. Yin T. Liu Z. Ming D.. (2013). A hybrid BCI speller paradigm combining P300 potential and the SSVEP blocking feature. J. Neural Eng. 10, 026001. 10.1088/1741-2560/10/2/026001 23369924

[B74] Xu M. Wang Y. Nakanishi M. Wang Y. T. Qi H. Jung T. P.. (2016b). Fast detection of covert visuospatial attention using hybrid N2pc and SSVEP features. J. Neural Eng. 13, 066003. 10.1088/1741-2560/13/6/066003 27705952

[B75] Yin E. Zeyl T. Saab R. Chau T. Hu D. Zhou Z.. (2015). A hybrid brain–computer interface based on the fusion of P300 and SSVEP scores. IEEE Transac. Neural Syst. Rehabil. Eng. 23, 693–701. 10.1109/TNSRE.2015.2403270 25706721

[B76] Yin E. Zhou Z. Jiang J. Chen F. Liu Y. Hu D.. (2013a). A novel hybrid BCI speller based on the incorporation of SSVEP into the P300 paradigm. J. Neural Eng. 10, 026012. 10.1088/1741-2560/10/2/026012 23429035

[B77] Yin E. Zhou Z. Jiang J. Chen F. Liu Y. Hu D.. (2013b). A speedy hybrid BCI spelling approach combining P300 and SSVEP. IEEE Transac. Biomed. Eng., 61, 473–483. 10.1109/TBME.2013.2281976 24058009

[B78] Yin E. Zhou Z. Jiang J. Yu Y. Hu D. (2014). A dynamically optimized SSVEP brain–computer interface (BCI) speller. IEEE Transac. Biomed. Eng., 62, 1447–1456. 10.1109/TBME.2014.2320948 24801483

[B79] Zhang S. Han X. Chen X. Wang Y. Gao S. Gao X.. (2018). A study on dynamic model of steady-state visual evoked potentials. J. Neural Eng. 15, 046010. 10.1088/1741-2552/aabb82 29616978

[B80] Zhang Y. Xu P. Liu T. Hu J. Zhang R. Yao D.. (2012). Multiple frequencies sequential coding for SSVEP-based brain-computer interface. PloS ONE 7, e29519. 10.1371/journal.pone.0029519 22412829 PMC3295792

