# Supplementary material for: A novel multiple time-frequency sequential coding strategy for hybrid brain-computer interface
Source: Front Hum Neurosci. 2022 Jul 29;16:859259. doi: 10.3389/fnhum.2022.859259 (PMC9372511; doi:10.3389/fnhum.2022.859259)
Supplement: Supplementary file 1 [file Data_Sheet_1.pdf]

Supplement material

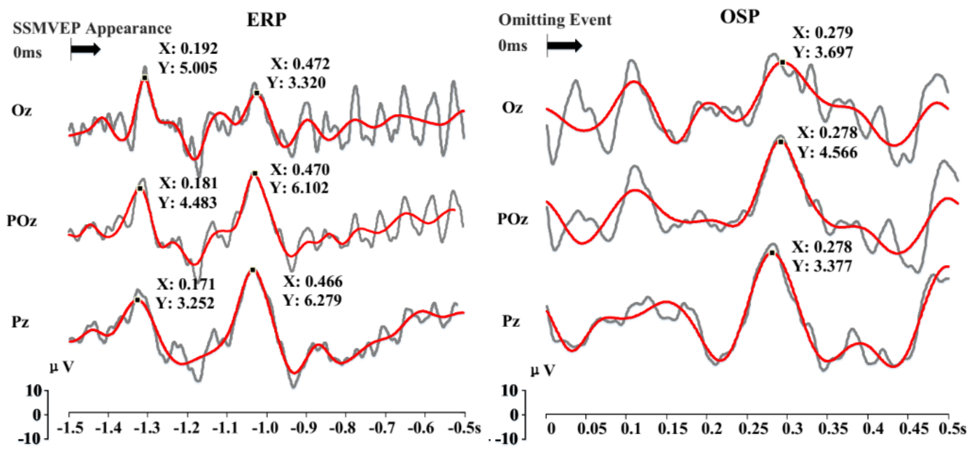

Fig. S1. The signal reconstructed based on the EMD method. The solid red line represents the reconstructed signal, and the solid grey line represents the averaged raw signal (64 trials). The top shows the response to the appearance of SSMVEP stimuli. The bottom shows the response to the omitting event.

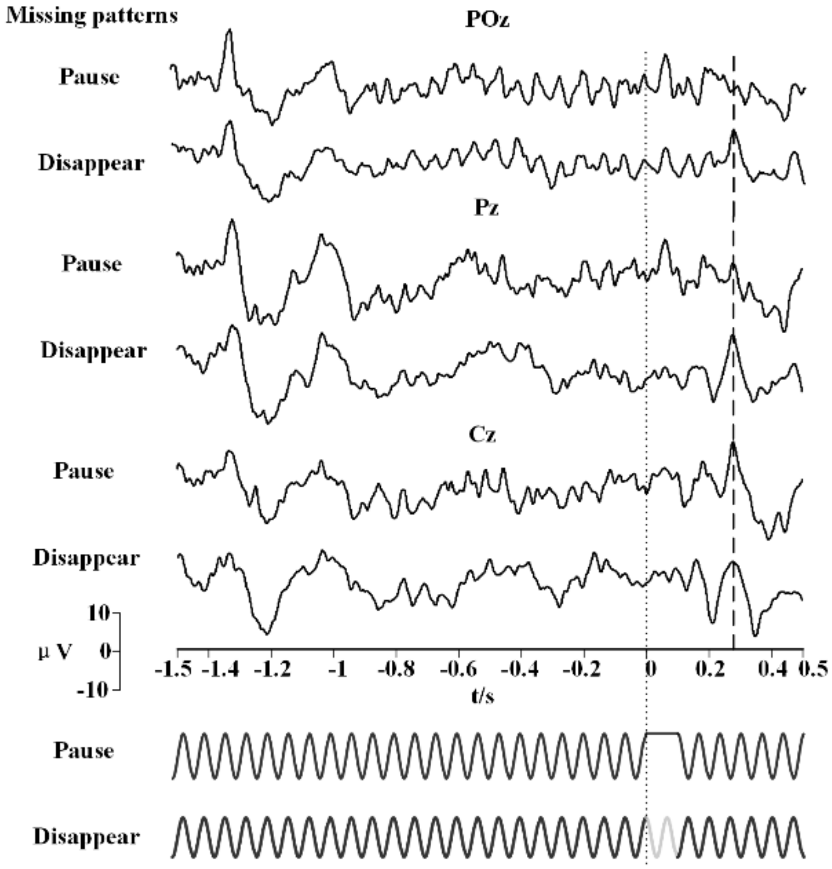

Fig. S2. Stimulus sequence of the stimulator and the typical EEG responses to the different missing patterns.

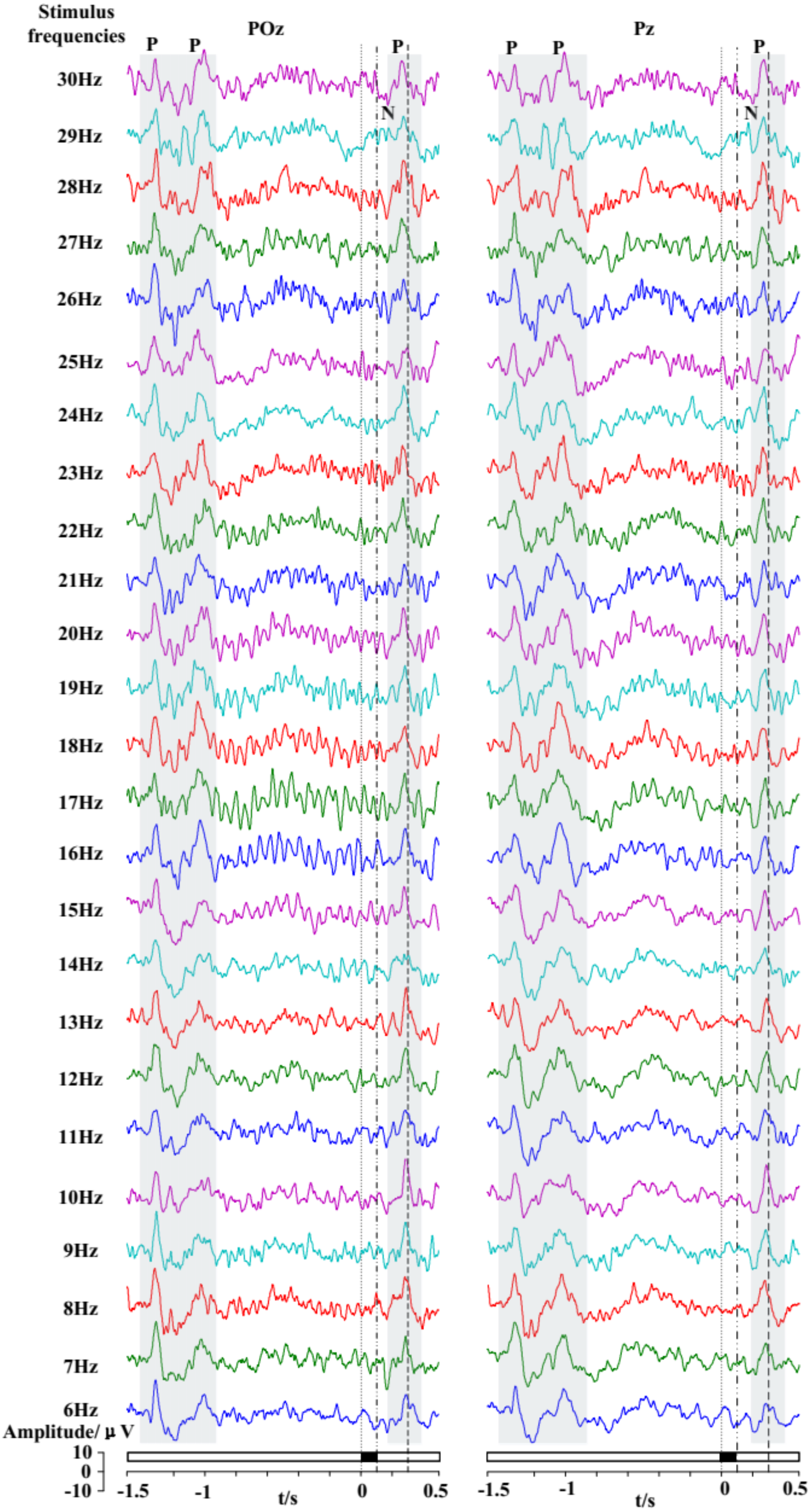

Fig. S3. Response to different frequencies was recorded by typical electrodes Pz and Poz. Gray-shaded area represent the primary features of ERP and OSP. ‘P’ and ‘N’ represents the dominent peaks.

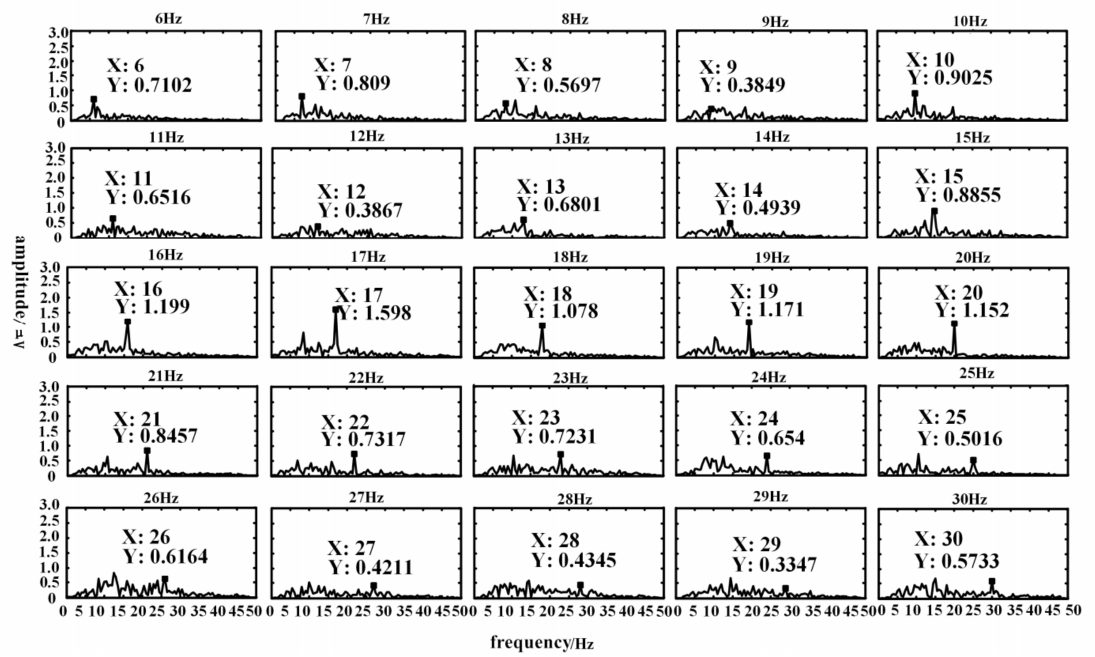

Fig. S4. The amplitude spectrum of SSVEP in different frequencies.

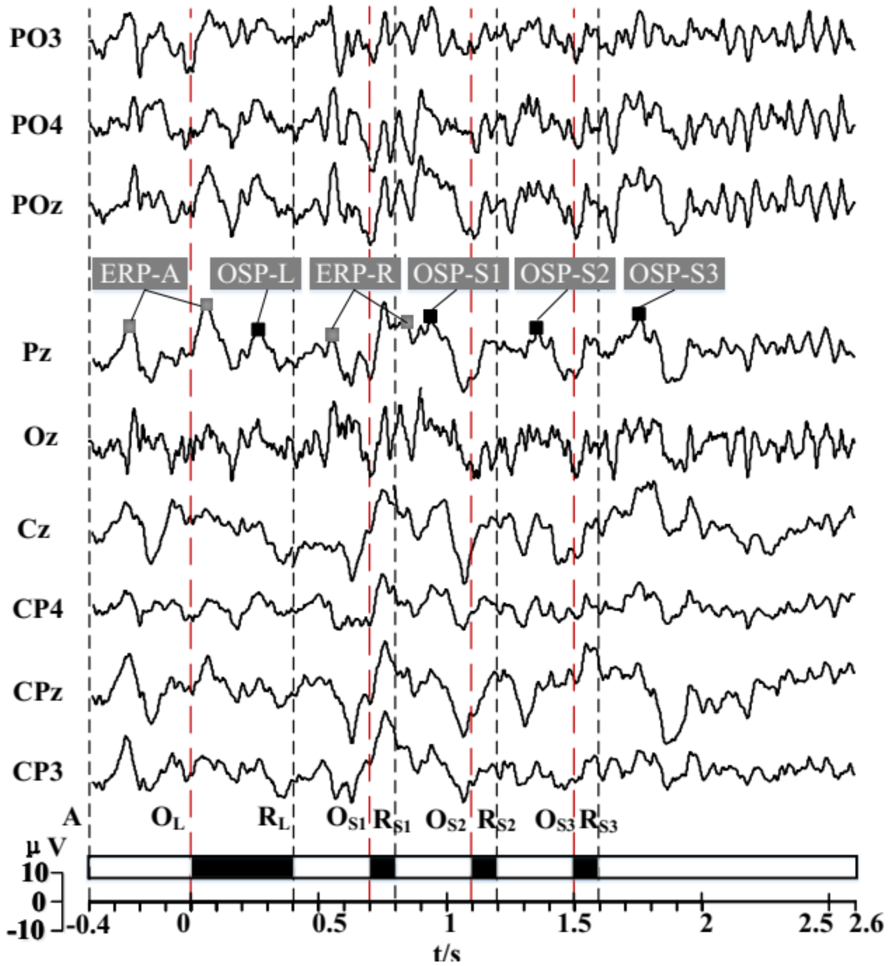

Fig. S5. The distribution of ERP and OSP in time domain. The time series of stimulus from part of channels.

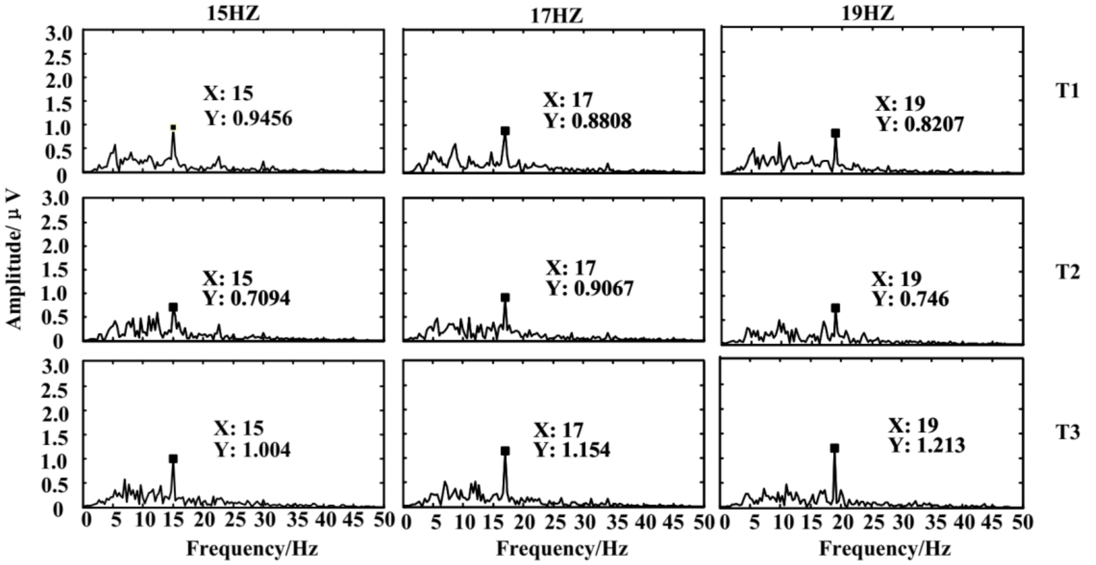

Fig. S6. The amplitude spectrum of the 9-targets experiment, SSVEP in different frequencies and different omision

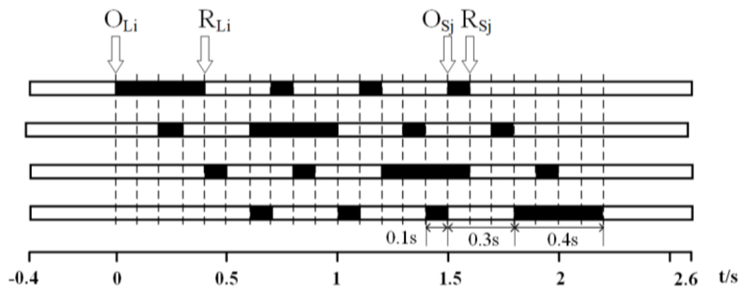

Fig. S7. The schematic diagram of the stimuli sequences. A kind of arrangement for omitting events was adopted in this study with 1 long omission (0.4S) and 3 short omission (0.1s). O_{L-i} represent the onset time of i th long omission, while the O_{Sj} represent the onset time of j th short omission. The R_{Li} (R_{Sj}) represents the end time of the i (j) th long (short) omission, or the time of reappearance of the SSVEP stimuli after the i (j) th omission.

Comparison between the Step-by-step recognition method (SSVEP + ERP) and the hybrid recognition method (template-based CCA).

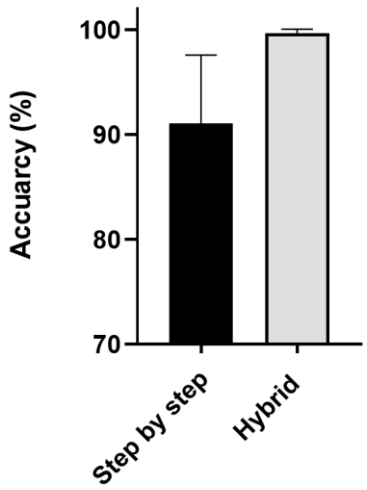

Fig. S9. Comparison of BCI performance on the the recognition algorithm based the Step-by-step recognition method (SSVEP + ERP) (91.05±6.56%) and the hybrid recognition method (template based CCA) (99.65±0.43%) based on data from offline MSTFC experiment.

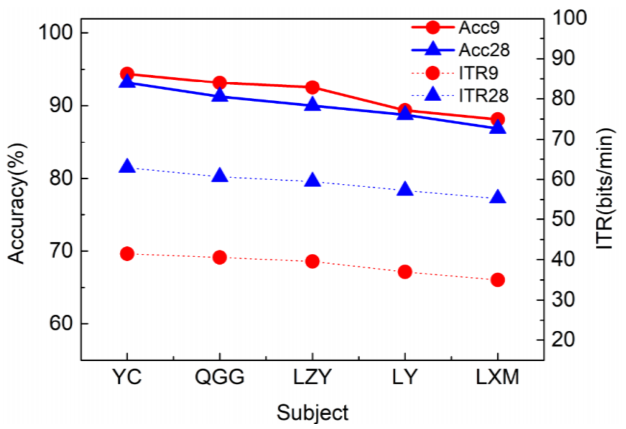

Fig. S8. Comparison of BCI performance on the same subjects between 9 stimulators and 28 stimulators.

Five subjects participated in further 28 targets paradigm. seven frequencies and 4 OSP patterns are used. These frequencies (10Hz, 15Hz, 16Hz, 17Hz, 18Hz, 19Hz, 22Hz and 24Hz) are selected from the result with apparent features of stimulation frequency experiments. Four stimulus sequences are used, as shown in Fig. S7

The 28 stimulator targets online task was implemented to explore the capacity of this novel paradigm in expanding the number of targets. The mean accuracy and standard deviation over five subjects were 90.00±2.38%, and the ITR (mean ± SD) over all subjects was 59.11±2.97 bits/min. As shown in Fig.S8, compared with the experiment result over the same subject on nine stimulators, ITR over 28 stimulators improved. At the same time, the accuracy decreased slightly with the increased number of stimulator targets.
